# Local Strain Engineering of Two-Dimensional Transition Metal Dichalcogenides Towards Quantum Emitters

**DOI:** 10.1007/s40820-024-01611-1

**Published:** 2025-01-08

**Authors:** Ruoqi Ai, Ximin Cui, Yang Li, Xiaolu Zhuo

**Affiliations:** 1 College of Electronics and Information Engineering, Shenzhen University, Shenzhen, 518060 People’s Republic of China; 2 School of Science and Engineering, The Chinese University of Hong Kong, Shenzhen, 518172 People’s Republic of China

**Keywords:** Two-dimensional transition metal dichalcogenides, Local strain, Excitonic behaviors, Quantum emitters

## Abstract

Two-dimensional transition metal dichalcogenides (2D TMDCs) have received considerable attention in local strain engineering due to their extraordinary mechanical flexibility, electonic structure, and optical properties. The strain-induced out-of-plane deformations in 2D TMDCs lead to diverse excitonic behaviors and versatile modulations in optical properties, paving the way for the development of advanced quantum technologies, flexible optoelectronic materials, and straintronic devices. Research on local strain engineering on 2D TMDCs has been delved into fabrication techniques, electronic state variations, and quantum optical applications. This review begins by summarizing the state-of-the-art methods for introducing local strain into 2D TMDCs, followed by an exploration of the impact of local strain engineering on optical properties. The intriguing phenomena resulting from local strain, such as exciton funnelling and anti-funnelling, are also discussed. We then shift the focus to the application of locally strained 2D TMDCs as quantum emitters, with various strategies outlined for modulating the properties of TMDC-based quantum emitters. Finally, we discuss the remaining questions in this field and provide an outlook on the future of local strain engineering on 2D TMDCs.

## Highlights

- Methods for creating the local deformation in two-dimensional transition metal dichalcogenides (2D TMDCs) are introduced.
- Modulations of local strain on their optical properties and excitonic behaviors are discussed.
- Quantum emitters based on strained 2D TMDCs and other applications are presented.

## Graphical abstract

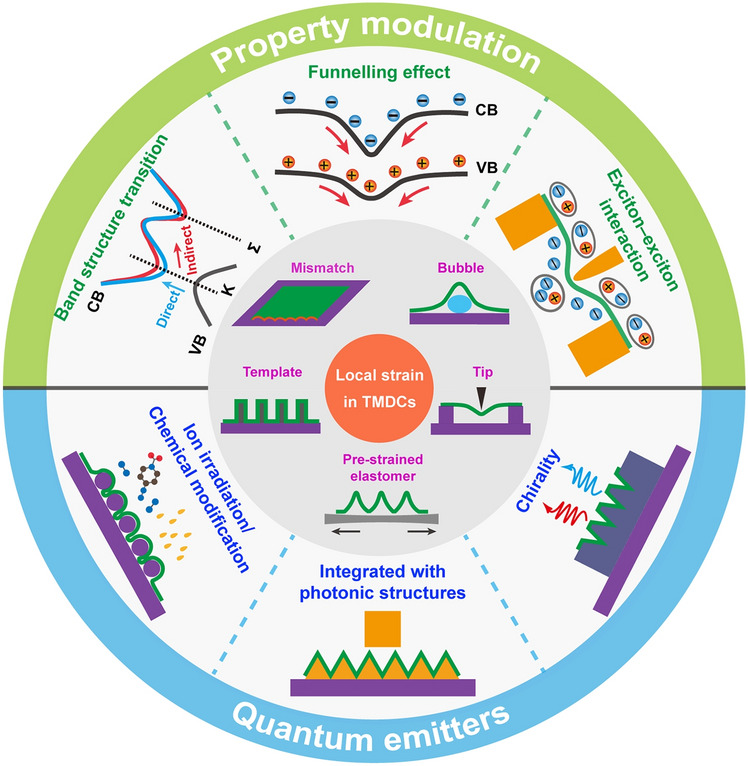

## Introduction

Two-dimensional transition metal dichalcogenides (2D TMDCs) have garnered significant attention in recent years due to their exceptional electronic [1, 2], optical [3, 4], and mechanical properties [5]. The atomically thin nature of 2D TMDCs gives rise to a strong quantum confinement effect, which confines photons and electrons within the planar dimensions. This confinement enables a diverse range of distinct phenomena, such as direct-to-indirect bandgap transitions [6, 7], rich excitonic complexes [7, 8], and valley pseudospin [9]. These unique properties of 2D TMDCs make them promising candidates for a wide range of applications, including high-performance electronics [10], optoelectronics [8], and energy storage [11].

The extraordinary flexibility and mechanical properties of 2D TMDCs enable them to tolerate significant structural curvature and accommodate large deformations in both the in-plane and out-of-plane directions. Specifically, 2D TMDCs can withstand up to 10% strain, which is an order of magnitude higher than their bulk counterparts [12–15]. As morphology deformation occurs, lattice distance and symmetry are altered, leading to the tuning in electronic band structures. This in turn modulates the optical properties and excitonic behaviors of 2D TMDCs [16, 17]. For example, a direct-to-indirect bandgap transition has been detected in monolayer MoS_2_ under tensile strain in uniaxial or biaxial directions [18, 19]. Out-of-plane deformation can introduce a non-uniform strain gradient in 2D TMDCs, enabling novel phenomena such as exciton funnelling [20, 21] and exciton-exciton interactions [22, 23]. The formed local excitons exhibit non-classical quantum emission, whose positions and emission characteristics are deterministically controlled by local strain fields [24–27]. The energies of emitted photons can be easily adjusted by strain field [17]. These characteristics make 2D TMDCs competitive candidates for the construction of single-photon light sources [28, 29].

A high-quality single-photon source is one of the essential building blocks for quantum optical applications, which can guarantee the security of quantum communications and minimize errors in quantum computation and simulation. 2D TMDC-based single-photon light sources have been linked to defects and highly confined strain fields [30, 31]. Integrating TMDCs-based single-photon emitters into an optical cavity or a waveguide is advantageous for enhancing single-photon coherence and brightness [32–36]. Implementing ion irradiation or chemical modification has proven to be a powerful method for optimizing quantum emitter performance [30, 37]. The determinacy of strained position [26, 38], the flexibility of integration [27, 39–41], and the versatile material library [42–44] render 2D TMDCs with unparalleled potential in quantum optical technologies compared to other material systems.

In this review, we present recent advancements in introducing local strain into 2D TMDCs, the resulting phenomena, and their applications. Representative approaches such as atomic force microscope (AFM) tips, pre-strained elastomer substrates, and integration of templated structures are discussed. The effects of local strain engineering on the optical properties of 2D TMDCs, especially the strain-induced excitonic behaviors, are discussed. Then, we give a brief overview of the applications for the 2D TMDC-based quantum emitters and the strategies for achieving single-photon quantum emitters. The challenges and prospects of this field are discussed at the end.

## Creation of Local Strain in 2D TMDCs

### Pre-Strained Elastomer Substrates

**Fig. 1:**
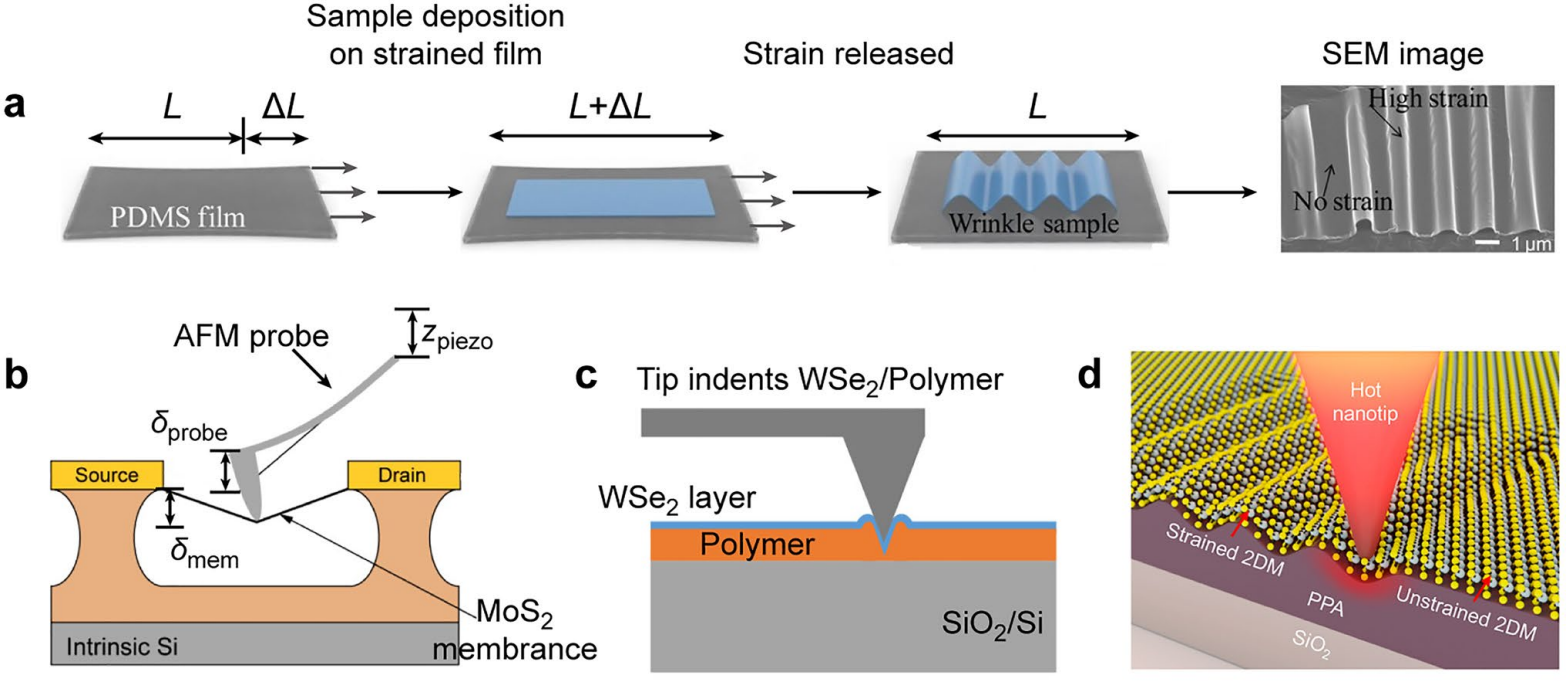
Creating local strain with pre-strained elastomer substrates and nanotips. **a** Schematic diagram showing the wrinkles formed in WSe_2_ layers through buckling-induced delamination. The last image is a representative scanning electron microscope (SEM) image of the obtained wrinkle structures. *L* represents the original length of substrates. Δ*L* is the change in the length variation of the elastomeric substrates relative to their original length during the stretching process. Reproduced with permission from [46]. Copyright 2017 American Chemical Society. **b** Setup for measuring the electrical properties of a suspended MoS_2_ membrane under strain applied by an AFM tip connected to a piezo scanner. The vertical displacement of the scanner (*δ*_piezo_) induces deformation in the centrally located MoS_2_ membrane. The deflection of the cantilever is *δ*_probe_. The vertical displacement of the membrane is *δ*_mem_. Reproduced with permission from [51]. Copyright 2015 American Chemical Society. **c** An AFM tip applying local force on a WSe_2_/PMMA layer. Reproduced with permission from [52]. Copyright 2019 American Chemical Society. **d** Schematic of strain deformation of 2D materials using t-SPL technique. Reproduced with permission from [53]. Copyright 2020 American Chemical Society

The elastic modulus mismatch between 2D TMDCs and substrates often leads to the presence of inhomogeneous strain within TMDCs [45, 46]. This strain distribution is further influenced when transferring 2D TMDCs onto pre-stretched elastomeric substrates (polydimethylsiloxane, PDMS) and releasing them [45–47]. The redistribution of strains triggers the delamination of 2D TMDCs from the underlying substrate, thereby forming periodic wrinkle structures (Fig. 1a) [46]. The efficiency of strain transfer increases with the mismatch in the elastic modulus and the enhancement of interfacial adhesion between the 2D TMDC and the substrate [48]. According to linear elastic theory, the local strain at the wrinkle peaks is proportional to the wrinkle amplitude and inversely proportional to the Poisson's ratio of the 2D TMDCs [49, 50]. However, the non-uniform wrinkle formation caused by the interfacial adhesion usually limits the ability to precisely control the local strain distribution. For the exfoliated TMDCs with limited scale, the overall yield of wrinkled 2D TMDCs is relatively low. The formed wrinkles tend to collapse due to the low stiffness of monolayer or bilayer TMDC systems [49], hindering the feasibility of this method for ultrathin 2D material systems.

### Nanotip-Induced Deformation

AFM tips, with their nanoscale curvature radius, allow for the generation of highly concentrated stress up to several gigapascals (GPa) and strain gradients of 10^8^ m^–1^ when in contact with 2D TMDCs [54, 55]. This method enables precise control, nanoscale spatial resolution, and real-time monitoring of the material's response, and therefore has been widely applied in prior works [56–59]. The excitonic drifting behavior of the strained TMDCs can be visualized by combining the AFM measurement with tip-enhanced Raman/photoluminescence (PL) spectroscopy [23, 60]. The reversible strain can be introduced into the TMDCs on grid substrates by using the AFM tip [61]. When the AFM tip is applied on the central region of the monolayer MoS_2_, the film exhibits a concave shape under the compressive strain. In contrast, near the film edges, the monolayer MoS_2_ displays a convex shape under tensile strain. However, the existence of the grid substrates usually constrains the deformation in the TMDC layers. To increase the deformation degree, one possible method is to press the AFM tip against TMDC films suspended over the grid structures [21, 22] or within the gap region between two adjacent structures [23, 51]. The deformation magnitude in the suspended MoS_2_ layer (*δ*_mem_) can be monitored and regulated at the same position, achieving maximum deformation magnitude of 33 nm (Fig. 1b) [51]. AFM tips can also induce a permanent form of local strain when the TMDC layers are placed on an elastic substrate, such as PDMS and polymethyl methacrylate (PMMA) films [52, 56]. In this approach, the deformation of the polymer substrate induces tensile strain in the overlying TMDC layer. The strong adhesion between the TMDC and substrate prevents relaxation, resulting in permanent spatially local strain profiles in the TMDC films (Fig. 1c) [52]. Polymers like PMMA and PDMS exhibit well-characterized stress–strain relationships, allowing for a reliable correlation between the applied force and the resulting permanent indent depth and shape. The capability of precise force control combined with the predictable response of the polymer materials ensures the high repeatability and reliability of this strain-engineering approach for 2D material/polymer composites. However, the high indentation force could potentially introduce damage to the TMDC layers. It’s still a challenge to scale up the strain engineering of TMDC materials with industrially relevant sizes and throughputs by using AFM-based methods.

Thermal scanning probe lithography (t-SPL) provides an alternative approach to overcome some of these limitations [53, 62, 63]. The t-SPL uses a heated nanotip to locally induce chemical changes or physical deformations in a polymer, allowing for the direct nanopatterning in TMDC layers (Fig. 1d) [53]. This approach allows for more uniform and controllable deformation patterns to be created across larger areas [64–66]. The moderate temperature used in t-SPL preserves the structural and chemical integrity of the 2D TMDCs.

### Bubbles

**Fig. 2:**
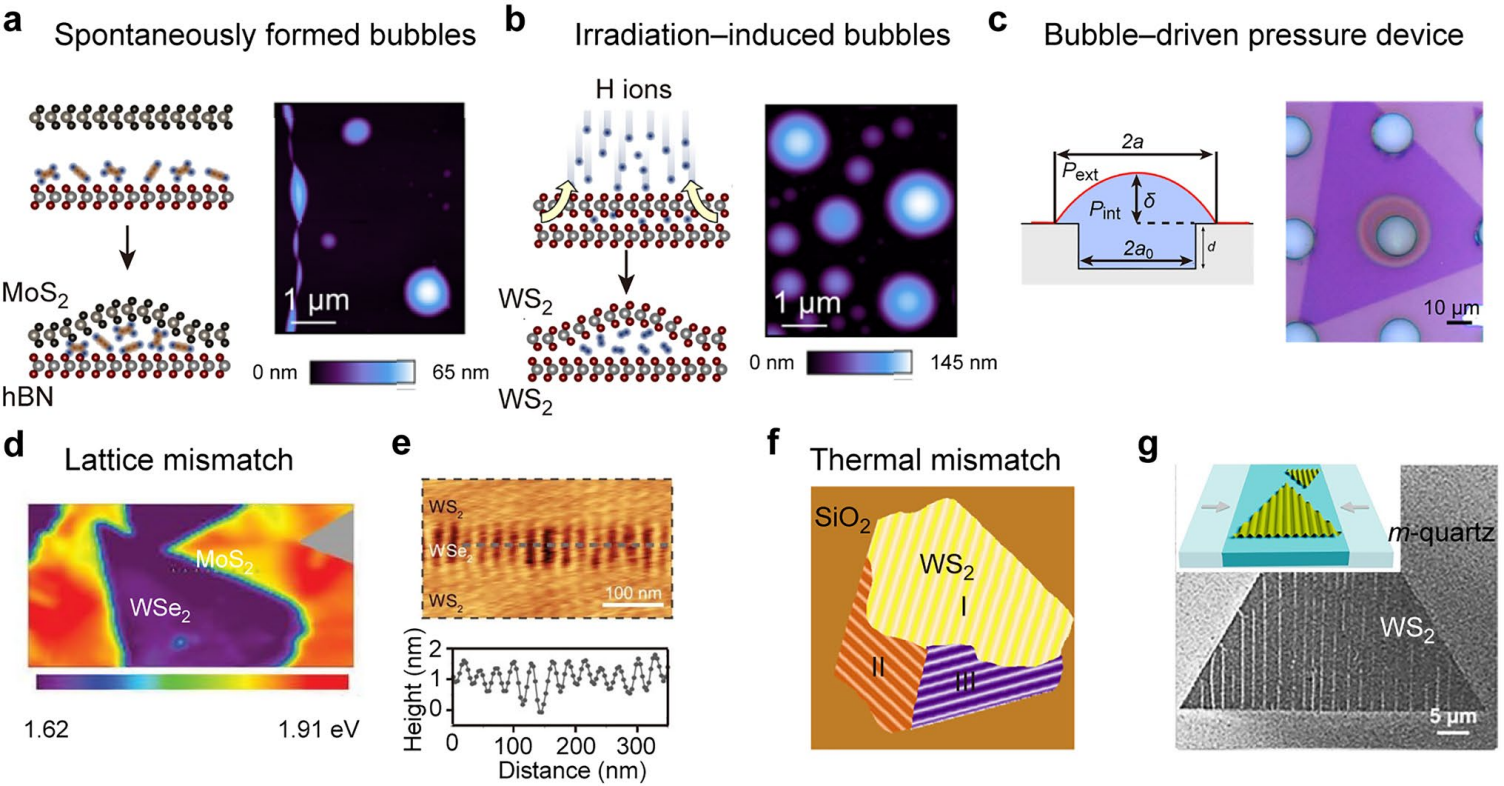
Local strain induced by lattice and thermal mismatch and bubbles. **a** Spontaneously formed bubbles. Left: Schematic illustration of bubbles fabricated by the spontaneous process. Right: AFM image for the bubbles produced in a MoS_2_/hBN vertical heterostructure. Reproduced with permission from [72]. Copyright 2021 American Physical Society. **b** Irradiation-induced bubbles. Left: Schematic illustration of bubbles fabricated with ion irradiation. Right: AFM image for the bubbles produced in a WS_2_/WS_2_ homostructure. Reproduced with permission from [72]. Copyright 2021 American Physical Society. **c** Bubble-driven pressure device. Left: Schematic diagram of a bubble-driven device by utilizing pressure difference. The chamber pressure is *P*_int_, and the external pressure is *P*_ext_ = 1 atm. The bubble radius and height are represented by *a* and *δ*, respectively. *a*_0_ represents the radius of the cylindrical microcavity. Right: Bright field optical microscope image of a formed bubble. Reproduced with permission from [83]. Copyright 2017 American Chemical Society. **d** PL mapping for the lateral WSe_2_/MoS_2_ heterostructures with PL peak variation in MoS_2_ region. Reproduced with permission from [87]. Copyright 2015 Springer Nature. **e** Lattice mismatch-induced ripples. Top: Representative AFM image of WS_2_/WSe_2_ superlattices. Bottom: Height profile extracted from the dashed line in the top AFM image. Reproduced with permission from [88]. Copyright 2018 AAAS. **f** Three rippling domains marked by I, II, and III induced by thermal mismatch in CVD-grown WS_2_ flake during cooling. Reproduced with permission from [89]. Copyright 2021 American Chemical Society. **g** SEM image for the WS_2_ wrinkles formed on *m*-quartz substrates. Inset schematic shows the thermal mismatch-induced strain distributing in a specific orientation. Reproduced with permission from [90]. Copyright 2021 American Chemical Society

The generation of bubbles in 2D TMDCs can be described as either spontaneous formation or deliberate creation. By transferring a 2D TMDC layer onto a substrate or another TMDC layer, the entrapment and accumulation of contaminants at the interface can help the generation of bubbles (Fig. 2a) [67–72]. These bubbles are spontaneously formed with random size and shape [72–74]. By submerging 2D materials and their substrates in a liquid environment, the spontaneous formation of bubbles can be facilitated with the existence of cracks, defects, and interlayer galleries in the 2D materials [75, 76]. Another approach to fabricate TMDC-based bubbles involves ion irradiation [77]. By exposing to low-energy H ions, the topmost or several layers of the TMDCs can be penetrated and the H_2_ gas is thus accumulated in the interlayers [78–80]. The delamination and bulging of the topmost TMDC layer contribute to the formation of H_2_-filled bubbles (Fig. 2b) [72]. The formed bubbles possess varying sizes ranging from a single atom [81] up to few microns [82]. To achieve a precise array of bubbles with controlled size and positioning, a H-opaque masking layer is pre-deposited on TMDC materials, and micrometer-scale openings are then fabricated on the masking layer before irradiation [77, 78]. This patterning procedure represents a tool for selective proton penetration and local hydrogen bubble formation. By optimizing the irradiation procedure, the bubble homogeneity and size distribution can be further improved. Bubbles can also be intentionally fabricated by transferring the 2D TMDCs onto a pre-patterned substrate with micro-cavities or holes (Fig. 2c) [83]. By adjusting the pressure difference between the inside and outside of the 2D TMDCs, the top layer materials can be precisely controlled to bulge. As shown in Fig. 2c, a MoS_2_ layer is suspended over the microcavities for the generation of a pressure chamber with gas introduction. The lateral dimensions, height, and shape of the deliberately created bubbles can be varied through the appropriate design of supported structures and pressure change. Apart from the formation of local strain, these bubbles contain complex compositions, such as aggregated gases and adsorbed pollutants, providing a favorable platform for studying the interfacial chemical processes between 2D materials and other substances at the nanoscale. Recent studies have demonstrated that the bubble-induced strain can engineer the graphene electronic states through the generation of a pseudo-magnetic field. Experimentally, Levy et al. analyzed the highly strained nanobubbles that generate when graphene is grown on a platinum (111) surface [84]. These bubbles exhibit the formation of Landau levels under strain-induced pseudo-magnetic fields exceeding 300 Tesla. The intensity of the pseudo-magnetic field scales with the magnitude of strain gradient, while the spatial distribution of the pseudo-magnetic field varies with the geometry of the bubble. Benefiting from the advancement of bubble manufacturing technology, the location, size, and shape of nanobubbles can be tuned by adjusting the stimulus bias of the AFM tip, enabling the generation of the well-designed pseudo-magnetic field in graphene [85]. In contrast to graphene, TMDC monolayers possess complex electronic structures and orbital interactions. The straightforward pseudo-magnetic field model for the strained graphene cannot be simply extend to the deformed TMDC monolayer. As a unique member of the TMDC family, Re-doped MoTe_2_ exhibits a ~ 3 Tesla pseudo-magnetic field in the presence of a small strain [86]. This strain-induced pseudo-magnetic field can generate quantized Landau levels, making it detectable in experiments.

### Lattice and Thermal Mismatch

Lattice mismatch-induced strain is inherent in the chemical vapor deposition (CVD) or epitaxial growth of 2D TMDCs and their heterostructures [48, 91, 92]. In heteroepitaxy, where epitaxial occurs between different materials, the lattice mismatch can induce lattice distortions at the interface [93, 94] or the formation of compressive or tensile strain in the epitaxial layer [87]. For example, MoS_2_ can epitaxially grow on the edges of WSe_2_ despite a large lattice mismatch, producing the lateral WSe_2_/MoS_2_ heterostructures [87]. The observed PL energy in MoS_2_ within the heterostructures, ranging from 1.79 to 1.91 eV at different locations (Fig. 2d), reflects the local strain distribution in the MoS_2_ domains. In another work of WS_2_/WSe_2_ heterostructures by Xie et al*.* the MoS_2_ region adjacent to WSe_2_ exhibits a tensile strain of 1.59 ± 0.25%, while the MoS_2_ areas further away from WSe_2_ show a compressive strain of 1.1 ± 0.18% [88]. This large difference in strain indicates the possibility of using monolayer TMDCs for straintronics applications. In addition, the lattice mismatch-induced strain can be released by the formation of out-of-plane ripples. By adjusting the dimensional ratio between WS_2_ and WSe_2_, the strain gradient can be precisely controlled. When the width of WSe_2_ is smaller than 320 nm, the compressive strain can be released by forming out-of-plane ripples with a periodic distribution of 1–2 nm (Fig. 2e). The periodic ripples cannot extend across the WSe_2_ when the width of WSe_2_ exceeds 320 nm. The strain relaxation through wrinkle formation is beneficial for maintaining the integrity and flatness of the overall superlattices.

Apart from lattice mismatch-induced local strain, the difference in thermal expansion coefficients (known as the thermal mismatch) can also induce strain between 2D TMDCs and their underlying substrates [90, 95–97]. This thermal mismatch-induced strain can be either compressive or tensile, which is dependent on the thermal expansion or contraction. Several studies have successfully detected thermal mismatch-induced strain in 2D TMDCs grown on various substrates, including mica [95], sapphire [98], and silicon oxide/silicon (SiO_2_/Si) [96, 98, 99]. Rapid cooling of as-grown WS_2_ flakes on SiO_2_ substrates can introduce rippling structures with isotropic compressive strain (Fig. 2f) [89]. The resulting wrinkle structures exhibit zigzag-orientated rippling domains that can be precisely manipulated by controlling AFM scanning. Moreover, anisotropic strain can be generated by utilizing quartz *m*-plane substrates with anisotropic thermal expansion coefficients (Fig. 2g) [90]. The size of the obtained wrinkle can be finely tuned below 100 nm by adjusting the quenching temperature. This uniaxial deformation in the wrinkled regions can result in the anisotropic Raman response and enhance second harmonic generation emissions. TMDC wrinkles produced by the thermal mismatch possess the key advantage in their capability to be produced on a rigid substrate at a large scale without requiring transfer. By selecting a substrate with a specific thermal expansion coefficient mismatch, the directionality of the formed local strain can be engineered. However, the complex folding, and collapse of the wrinkles are unavoidable during the fabrication process, which limits the uniformity and reproducibility of local strain produced in TMDC materials [100].

### Patterned Templates

Integrating 2D TMDCs with patterned templates provides another effective strategy for precise and deterministic strain engineering. This approach enables the control of local exciton behavior and offers flexibility in structural design. Patterned templates can be fabricated using different methods, including chemical synthesis, electron/ion beam lithography, optical lithography, electron beam etching, and ion beam sputtering [38, 48, 101]. Both individual nanostructures and designed arrays can serve as templates for inducing local strain [102, 103].

**Fig. 3:**
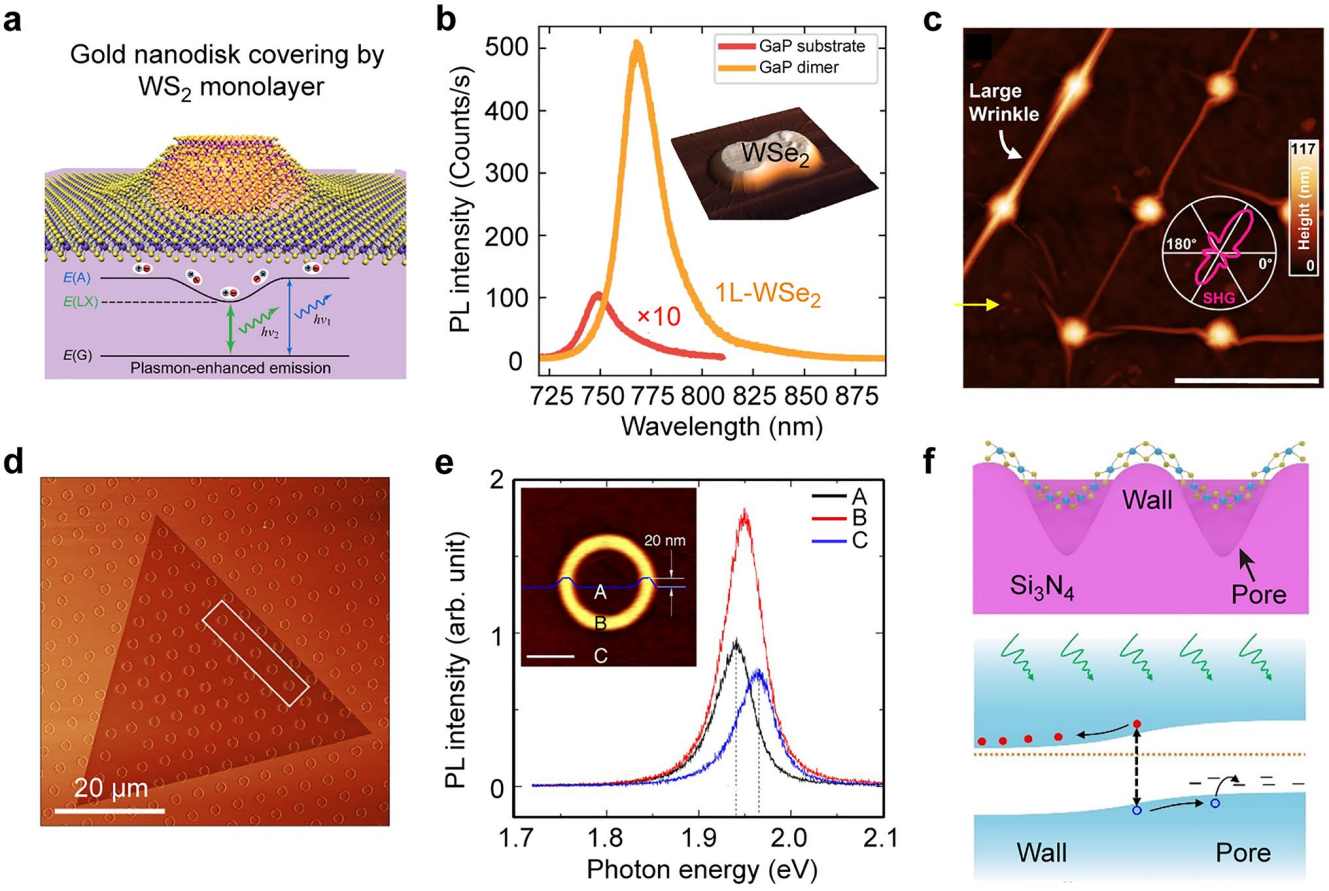
Local strain induced by patterned templates. **a** Schematic showing a gold nanodisk covered with a monolayer WS_2_ and schematic showing the working principle of the syructures. The *E*(A), *E*(G), and *E*(LX) represent the energy levels of the neutral excitons, ground state excitons, and strain-localized excitons, represpectively. Reproduced with permission from [107]. Copyright 2022 American Chemical Society. **b** PL spectra obtained from 1L WSe_2_ placed on GaP substrate (red) and GaP nanoantennas (orange). Inset image: AFM image of a monolayer WSe_2_ on the top of GaP nanoantennas. The strain is localized within the edges of the nanoantennas. Reproduced with permission from [116]. Copyright 2019 Springer Nature. **c** AFM image of monolayer WSe_2_ wrinkles on the nanocone array. The scale bar is 500 nm. Reproduced with permission from [103]. Copyright 2024 Springer Nature. **d** AFM phase image of monolayer WS_2_ deposited on the pre-patterned donut array. **e** PL spectra obtained from the positions A–C shown in inset image. Inset image: AFM image collected from a single donut covered with a layer of WS_2_. The height of the donut is 20 nm. The blue line represents the height profile of the donut. Reproduced with permission from [120]. Copyright 2019 AAAS. **f** Top: Schematic illustrating the cross-section of the monolayer WS_2_ grown on the suspended Si_3_N_4_/Si substrate. Bottom: Band structure of the monolayer WS_2_ between the pore and wall. Reproduced with permission from [121]. Copyright 2023 American Chemical Society

Diverse nanophotonic structures, from dielectric nanoantenna to plasmonic resonators, have been widely employed to induce local strain in 2D TMDCs [104–107]. Noble metal nanostructures are the promising templates for controlling strain deformation and distribution in 2D TMDCs because of their diverse shapes, tunable sizes, and uniform distribution [108, 109]. The combination of plasmonic nanostructures and 2D TMDCs can also greatly enhance the emission of local excitons through the excitation of localized surface plasmon resonances [25, 106, 107]. Numerous metallic stressors, such as silver nanocones [32], silver nanowires [40], gold rectangles [110], gold nanodisks [107], and gold nanostars [111], have been used to introduce local strain and further exploit the Purcell effect for enhanced emission in TMDCs. For example, the strain-localized excitons in a WS_2_ monolayer have been observed when covering individual gold nanodisks or nanorods at room temperature, leading to remarkably enhanced emission with a redshift of to 200 meV (Fig. 3a) [107]. The emission properties of the strain-localized excitons can be tailored by adjusting the size of the nanodisks. This structural design combines effective strain localization with spatial and spectral engineering of plasmonic field enhancement. However, plasmonic nanostructures suffer from large non-radiative losses due to the interband transitions [112], resulting in Joule heat generation that is detrimental to both the nanostructures and their applications [113, 114]. To overcome this problem, the use of dielectric nanostructures is proposed as a complementary approach [102, 115–117]. High-refractive-index dielectric nanostructures offer a lossless operation [112] and can support Mie resonance modes, producing strong electric and magnetic field enhancements [118]. These properties make them excellent candidates for enhancing the emission of quantum emitters. For example, placing WSe_2_ on a SiO_2_ nanopillars can singificantly boost their single-photon emission intensity, using the SiO_2_ nanopillars as optical resonators and local strain introducers [38, 116, 117]. Additionally, high-refractive-index gallium phosphide (GaP) nanoantennas have been demonstrated to improve the quantum efficiency of emitters in 2D TMDCs [116]. The PL intensity of the emitter formed on GaP nanoantennas is 10^4^ times brighter compared to the WSe_2_ monolayer placed on planar GaP (Fig. 3b). In addition to using individual nanostructures as templates, arrayed structures defined by e-beam lithography or photolithography are also commonly employed to induce period strain into 2D TMDCs [26, 103, 119]. The integration of 2D TMDCs with these structural arrays offers significant advantages for constructing highly integrated and multifunctional devices. Local deformations on the patterned arrays often display highly inhomogeneous and therefore affect the emission properties of local excitons [26]. The direction and morphology of wrinkles on the WSe_2_ can be controlled by precisely tuning the geometric features of the arrayed substrates (Fig. 3c) [103]. In triangular arrays, the predominant wrinkle directions are 60°/240°, reflecting the symmetry of the substrate array (Fig. 3c). Further investigation of the relationship between deformation position and emitter center is crucial for promoting the large-scale production and practical application of such emitters.However, these methods typically rely on a polymer-assisted transfer process with mechanically exfoliated 2D TMDCs. The inherent defects and non-uniformity of the exfoliated flakes can lead to uncontrolled strain distribution, severely limiting the performance and reliability of devices based on 2D TMDCs.

Direct growth of 2D TMDCs on pre-patterned substrates is another important approach to introduce local strain [92, 120, 121]. The substrates with patterned structures are prepared first, followed by direct deposition of 2D TMDCs onto these pre-patterned structures. The TMDC can be grown conformally onto the surface of the underlying substrate. For example, monolayer WS_2_ has been successfully grown without wrinkles, folds, or suspended features on SiO_2_/Si substrates patterned with circular ring ("donut") structures (Fig. 3d) [120]. The resulting strain field exhibits a symmetric profile, with the highest strain region localized at the center of the donut structures (Fig. 3e). This local strain can accelerate the crystal growth in a specific direction, offering a strategy for engineering the growth direction and modulating the optoelectronic properties of 2D TMDCs. Another example is the phototransistor based on a partially suspended monolayer WS_2_ grown on a porous Si_3_N_4_/Si substrate, demonstrating a high responsivity and a fast response speed [121] comparable to those of the Si nanowire phototransistors [122]. Tensile strain gradients between the pore region and the surrounding WS_2_ film can lead to band bending of WS_2_ and form a built-in electric field (Fig. 3f) [121]. Under illumination, the built-in electric field effectively separates photogenerated carriers, resulting in high responsivity. Owing to the variety of available substrate morphologies, the direct growth technique holds great promise for enabling localized strain modulation. The direct growth of 2D TMDC on pre-patterned substrates offers a promising pathway to achieve controlled strain distributions, as well as scalable and reproducible fabrication of strained 2D TMDCs.

All the methods discussed in the manuscript can be used to create local deformation in 2D TMDCs. The characteristics of the induced strain vary with different methods whose technical requirements are unlike. To produce a periodic strain with wrinkled or rippled morphologies, researchers can introduce local strain on 2D TMDCs by using pre-stretched elastomer, which offers the advantage of easy operation. Using AFM techniques to induce indentation or transferring 2D TMDCs on patterned structures is beneficial for creating the local deformation with highly determine in both locations and magnitude. Additionally, the generation of bubbles by transferring 2D TMDCs suspended on structures can help realize control of the strain magnitude and location. To achieve large-scale strained samples, CVD methods can be used, such as directly growing 2D TMDCs on pre-patterned substrates or producing rippling and wrinkled structures in grown TMDCs through the thermal mismatch between substrate and material. Table 1 is provided as a reference for choosing the appropriate method for local strain generation.

**Table 1:** Summary of fabrication techniques for generating local strain into 2D TMDCs

Techniques	Materials	Substrates	Features	Properties modification	Adv. and Dis.	References
Pre-strained elastomer	ML/FL WSe_2_	PDMS	Wrinkles	1%–2% strain range; 120 meV reduction in indirect bandgap	Adv.: Easy to operation; creation for periodic strain Dis.: Limited sample size, easy collapse	[46]
	FL MoS_2_	PDMS	Wrinkles	0.2%–2.5% strain range; 90 meV reduction in indirect bandgap		[45]
	ML/FL WS_2_	PDMS	Wrinkles	1%–2% strain range; 110 meV reduction for indirect bandgap		[46, 49]
	MoS_2_/WSe_2_ heterobilayers	PDMS	Wrinkles	∼ 107 meV for the modulation of interlayer exciton bandgap		[123]
Nanotip-induced deformation	ML MoS_2_	SiO_2_ substrates	Reversible point deformation	Maximum 0.1% strain	Adv.: Precise and site-selective strain creation Dis.: Limited sample size, complex techniques	[61]
	ML WSe_2_	Suspended on TEM grid	Reversible point deformation	Bright exciton funnelling		[51]
	ML WSe_2_	PMMA	Permanent point deformation			[52]
	ML MoS_2_	PPA	Permanent point deformation by using a heated nanotip	∼ 180 meV bandgap modulation		[53]
Bubbles	ML MoS_2_	hBN	Spontaneous formation		Adv.: Simplifying experimental setup Dis.: Difficult to control of bubble size and distribution	[72]
	FL WS_2_	WS_2_	Ion irradiation			[72]
	ML MoS_2_	Suspended over the microcavities	Pressure difference		Adv.: Control of bubble position and size Dis.: Limited stability and applicability	[83]
Lattice mismatch	WSe_2_/MoS_2_ heterostructures	SiO_2_ substrates	Compressive or tensile strain in the epitaxial layer	1.59 ± 0.25% tensile strain in WSe_2_; 1.1 ± 0.18% compressive strain in WS_2_	Adv.: Periodic strain distribution Dis.: Local strain area distributed in specific positions and small in extent	[87]
	WS_2_/WSe_2_ heterostructures	SiO_2_ substrates	Ripples			[88]
Thermal mismatch	ML WS_2_	SiO_2_ substrates	Wrinkles	Isotropic compressive strain	Adv.: Large-scaled strain area Dis.: Limited strain manipulation	[89]
	ML WS_2_	*m*-quartz substrates	Wrinkles along one direction	Anisotropic strain		[90]
Patterned templates	ML WS_2_	Mechanical transferring on Au nanodisks	Deformation in the proximity of templated structures and wrinkles in tent region	Generation of local excitons and enhancement of their emission	Adv.: Precise and deterministic strain engineering; control of local exciton behaviors Dis.: Limited sample size	[107]
	ML WSe_2_	Mechanical transferring on GaP nanoantennas				[116]
	ML WSe_2_	Mechanical transferring on Au nanocone array				[103]
	ML WS_2_	Direct growth on pre-patterned trenches and donuts	Distribute along trenches and donuts with a symmetric profile		Adv.: Large-scaled sample size and stable strain deformation Dis.: Limited strain magnitude and high cost for the fabrication of pre-patterned substrates	[120]

FL: few layers, ML: monolayer, PMDS: polydimethylsiloxane, PPA: polyphthalaldehyde

## Local Strain Effect on Physical Properties of 2D TMDCs

Numerous studies have conducted the local strain effect on the electronic, optical, and magnetic properties of 2D TMDCs from experimental and theoretical prospectives. Strain-induced lattice and symmetry altering can tune electronic band structures and result in several intriguing phenomena, like exciton funnelling and anti-funnelling. Many theoretical studies have developed to promote the understanding of how strain modify multiple functionalities as well. In this section, we summarize the local strain effect on modulated physical properties of 2D TMDCs across excitonic behaviors, and optical, and magnetic properties.

### Modulated Excitonic Behaviors

Local strain can significantly change the electrical structure of 2D TMDCs and thus increase or decrease the bandgap. A sufficient magnitude of strain gives rise to a transition between the direct and indirect bandgap. This spatial variation in the energy landscape can cause the preferential movement of excitons towards specific regions, which is known as exciton funnelling or anti-funnelling effects. Exciton-exciton interaction occurs in the funnelling region, such as the exciton-to-trion conversion or hybridization of different excitonic states.

#### Bandgap Engineering

**Fig. 4:**
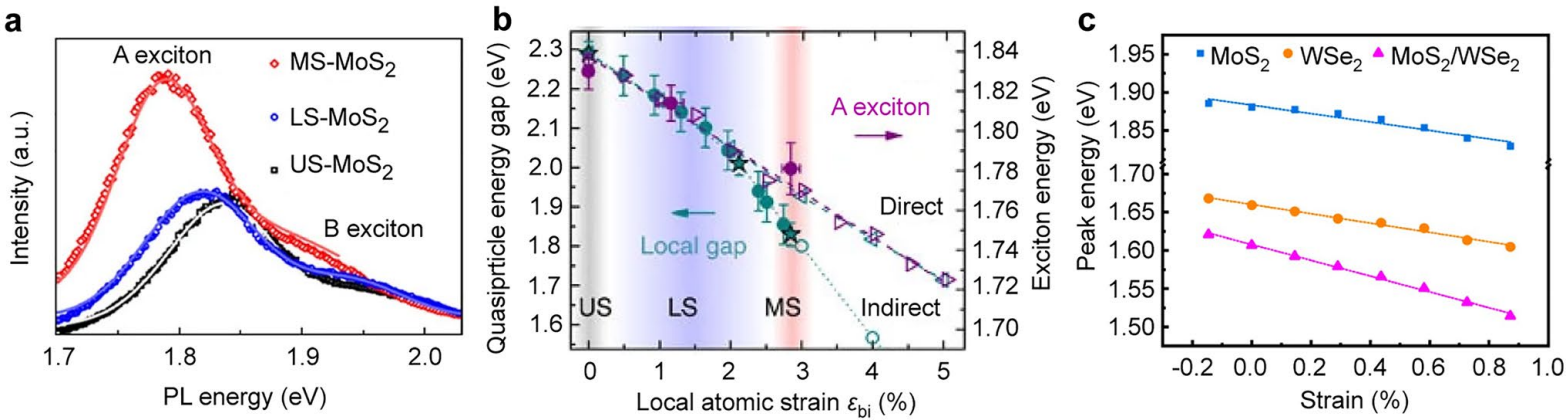
Local strain-modulated excitonic properties. **a** PL spectra collected from the most strained-MoS_2_ (MS-MoS_2_, red symbol), less strained-MoS_2_ (LS-MoS_2_, blue symbol), and unstrained-MoS_2_ (US-MoS_2_, black symbol). **b** Theoretical and experimental results for the exciton energy (magenta, right axis) and the quasiparticle energy (cyan, left axis). The hollow symbol represents theoretical data, and the solid symbol represents experimental data. Reproduced with permission from [130]. Copyright 2015 Springer Nature. **c** Deformation peak energy of the intralayer excitons in MoS_2_, WSe_2_ and the interlayer exciton in MoS_2_/WSe_2_ as a function of strain. The solid symbols represent the measured data. The dashed lines represent the linear fitting results. Reproduced with permission from [123]. Copyright 2021 American Chemical Society

2D TMDC materials, with their large surface-to-volume ratio and outstanding stretchability, serve as a good platform for tuning electronic structure and bandgap under an external strain field [17, 124]. Raman and PL spectroscopic techniques have been widely used to estimate the strain-induced changes in the band structure of 2D TMDCs [125–127]. Although many studies have introduced homogeneous strain on 2D TMDCs with elastic substrates for strain-induced band engineering [128, 129], the generation of local strain offer an effective way for modulating bandgap deterministically. Local strain has been successfully induced in monolayer MoS_2_ by transferring it onto SiO_2_ nanocones [130]. This results in a noticeable redshift of the A exciton, with energy changes of 18 and 50 meV for the less strained and most strained MoS_2_, respectively (Fig. 4a). A pronounced decrease in PL intensity of monolayer MoS_2_ has been detected due to the transition from a direct to an indirect bandgap with increased strain. Theoretical simulations further confirm a direct-to-indirect gap transition at 2% biaxial tensile strain (Fig. 4b) [130].

Apart from its impact on bandgap engineering in monolayer TMDCs, local strain also plays an important role in modifying interlayer excitonic band structures. Nam et al. have reported a Γ-Κ momentum-space indirect transition by inducing local strain in wrinkled vertical heterostructures on an elastomeric substrate [123]. Because of the misalignment of the crystallographic axes in MoS_2_ and WSe_2_, the effective oscillator strength of Κ-Κ transition would be weaker. For the strained MoS_2_/WSe_2_ heterostructures, the deformation potential of Γ-Κ transition is more sensitive than that of Κ-Κ transition, which agrees on the experimentally observed value. For the MoS_2_/WSe_2_ heterostructure, a large peak shift of 107 meV is achieved, twice that of the constitute intralayer excitons under the uniaxial strain, which is about 54 meV for WSe_2_ and 55 meV for MoS_2_ intralayer excitons (Fig. 4c) [123]. The PL intensity of interlayer excitons decreases with both increasing tensile and compressive strain. The interlayer coupling is weakened with the applied strain, leading to a reduction in the oscillator strength of the interlayer excitons and a decrease in the emission intensity.

#### Exciton Funnelling and Anti-Funnelling Effects

**Fig. 5:**
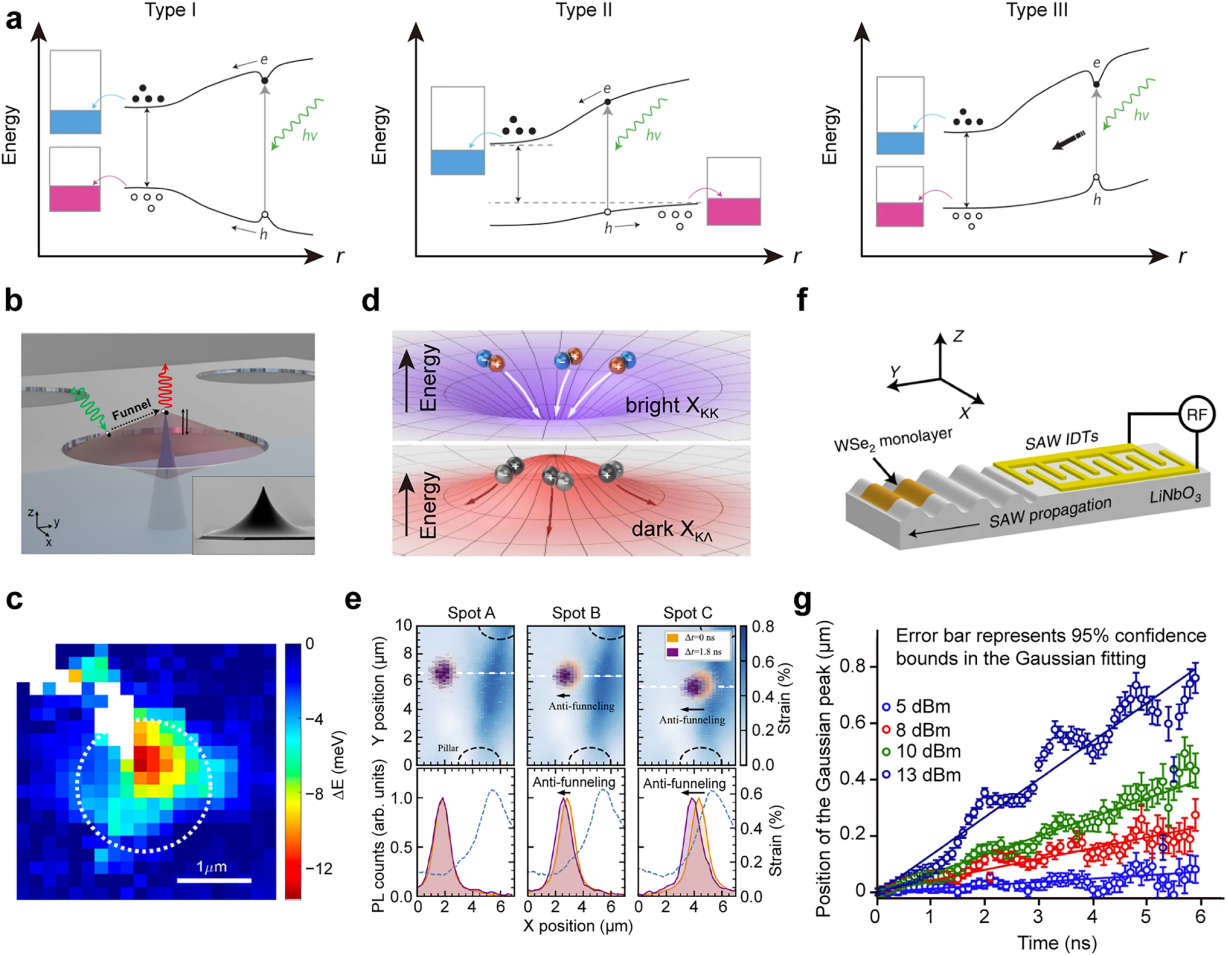
Exciton funnelling and anti-funnelling effects. **a** Three funnelling mechanisms originating from a different band bending, and exciton binding profiles for the strain-engineered 2D TMDCs. Reproduced with permission from [131]. Copyright 2012 Springer Nature. **b** Schematic showing dynamic control of exciton flux through a cantilever with a sharp tip applied on a WSe_2_ layer. The WSe_2_ layer is suspended on a transmission electron microscopy (TEM) grid. The inset SEM image shows the actual tip. **c** Spatial map of the free-exciton energy shift due to the indentation. The white dashed circle represents the region collected for the fitting analysis. Reproduced with permission from [21]. Copyright 2020 American Chemical Society. **d** Schematic illustrating the strain-induced funnelling of the bright exciton (top panel) and anti-funnelling of the dark exciton (bottom panel). The opposite energy shifts of X_ΚΛ_ and X_ΚΚ_ lead to reverse spatial energy gradients, giving rise to the propagation of dark exciton to the low-strain region. **e** Exciton anti-funnelling effect in strained WS_2_ monolayer. Top: Time-resolved PL collected from the strained WS_2_ region between two micropillars (black dash lines) at a delay time of 0 ns (orange) and 1.8 ns (purple) after the pulsed excitation. The blue map image corresponds to the measured strain distribution profile in the WS_2_ layer caused by the micropillars. Spot A: Excitation spot is far from a strain gradient, no exciton prepagation can be observed. Spots B and C: When the excitation spot is positioned close to a significant strain gradient, exciton anti-funnelling towards low-strain regions can be observed. Bottom: PL intensity profiles are collected along the horizontal dashed lines crossing the excitation spot in the top images. Reproduced with permission from [136]. Copyright 2021 Springer Nature. **f** Schematic illustrating the arrangement of a monolayer WSe_2_ transferred onto a SAW delay line. **g** Spatial evolution of the Gaussian exciton density peak as a function of time. The circles represent the exciton density peak extracted using Gaussian fitting. Different colors correspond to the data collected under different excitation powers. The error bars represent the 95% confidence interval of the Gaussian fit. Solid lines are the approximate linear fit. Reproduced with permission from [137]. Copyright 2022 Springer Nature

Local inhomogeneous strain can induce a spatial variation in the band energy landscape, leading to the preferential movement of excitons towards specific regions, known as exciton funnelling. Three funnelling mechanisms have been identified according to the exciton binding energy and the energy profiles of electrons and holes [131]. In the first mechanism (Type I), the energy level of photoexcited electrons continuously decreases while that of holes increases (Fig. 5a, left panel). Given sufficiently long lifetime, electrons and holes migrate separately to the central region where the energy of electron–hole pairs is lowest. The Type II mechanism relies on a weak binding energy of excitons, causing electron–hole pairs to separate due to a built-in field resulting from the difference in energy level reduction of electrons and holes (Fig. 5a. middle panel). If the exciton binding energy is strong enough despite the built-in field, the bounded electron–hole pairs undergo a downward shift towards the central region along the energy gradient created by external strain. This mechanism is classified as the Type III funnel (Fig. 5a, right panel). Exciton funnelling induced by strain has been demonstrated in various geometrical deformations of TMDCs, including wrinkled MoS_2_ [45], MoS_2_ nanobubbles [132], and textured MoS_2_ and WSe_2_ [130, 133, 134]. Recent studies have paid more attention on achieving deterministic exciton funnelling in a controlled and visualized way [21, 60, 135, 136]. For example, the exciton funnelling has been visualized on a suspended WSe_2_ monolayer by introducing a local strain with an AFM tip (Fig. 5b, c) [21]. The energy shift of free excitons in the indentation region reaches its maximum at the tip position, indicating that excitons are dragged away from the excitation region and recombined in the tip region of the highest strain. The spatial distribution of the exciton energy shift provides a visualization of the exciton funnelling and strain-induced bandgap modulation.

Contrasting the aforementioned funnelling effect, another intriguing phenomenon known as anti-funnelling effect has been observed in the behavior of dark excitons under local strain [136, 138]. This effect can be attributed to dark ΚΛ exciton, which possesses an opposite strain-induced energy variation compared to the bright excitons (Fig. 5d) [136]. Spatiotemporal exciton dynamics has revealed that dark excitons excited in highly strained regions migrate towards regions with lower strain (Fig. 5e). According to calculations, the energy of momentum-dark excitons increases with the applied strain, unlike bright excitons. A drift force induced by energy gradient can push the dark excitons towards regions of higher energy, leading to an anti-funnelling effect. The propagation distances for the dark excitons are comparable to the reported funnelling length of the bright excitons [21, 133]. In addition to the exciton spatial transport induced by static strain, Datta et al*.* have demonstrated that the dynamic strain can also direct the exciton transport by creating a traveling strain field on monolayer WSe_2_ (Fig. 5f) [137]. The exciton emission peak shows the spatial drift over time, while the direction of excitons is aligned with the propagation direction of the surface acoustic waves (SAW) propagation. As the radio frequency power increases, the spatial shift becomes more pronounced and exhibits periodic oscillations corresponding to the SAW period (Fig. 5g). The coupling between excitons and dynamic strain waves is influenced by various factors including intrinsic and extrinsic defect states, lattice disorders, substrate roughness, and scattering from charged and neutral impurities. This work provides valuable insights for achieving long-range exciton transport under ambient conditions.

#### Exciton-Exciton Interactions

**Fig. 6:**
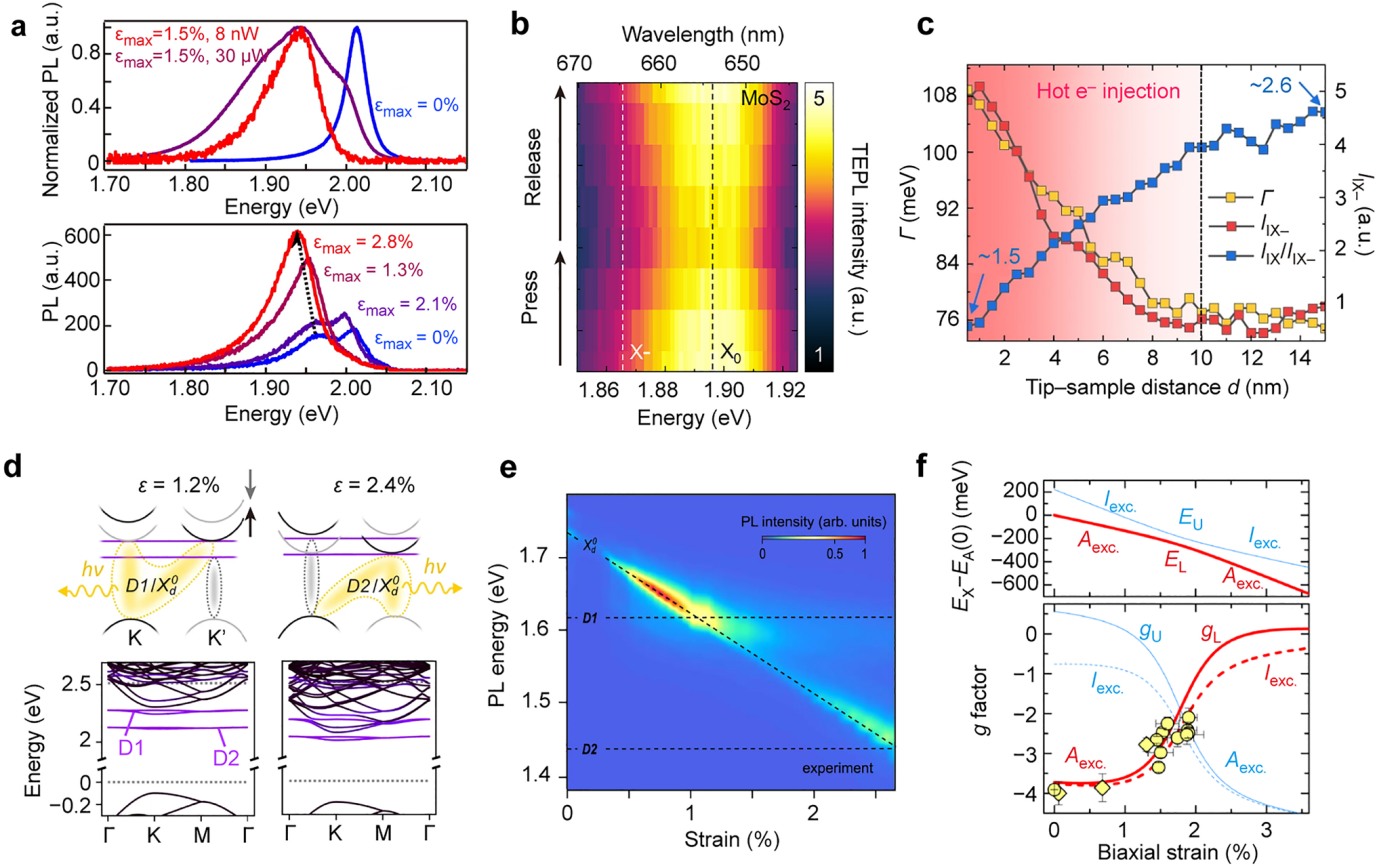
Exciton-exciton interactions. **a** Exciton-to-trion conversion of WS_2_ monolayer at various strain levels. Top: Strain- and power-dependent PL spectra of monolayer WS_2_ suspended over a hole. *ε*_max_ is the maximal strain reached underneath the AFM tip. The blue curve represents the PL spectrum collected from unstrained regions under an excitation power of 30 μW. The red and purple curves correspond to the PL spectra collected from the strained region (*ε*_max_ = 1.5%) under excitation power of 8 nW and 30 μW, respectively. Bottom: Strain-dependent PL spectra for the *n*-doped WS_2_ sample collected under different strains. Two PL peaks can be detected in the unstrained region, one of which locates at 1.965 eV corresponding to trions. Reproduced with permission from [22]. Copyright 2020 Springer Nature. **b** Tip-enhanced PL spectra collected from monolayer MoS_2_ under a series of dynamic strain. The white and black dashed lines correspond to the energy of neutral exciton (X_0_) and trion (X_–_). The dynamic strain is realized by pressing the gold tip onto MoS_2_ and subsequently releasing it from MoS_2_. Reproduced with permission from [23]. Copyright 2022 AAAS. **c** Evolution of the linewidth of interlayer trion (*Γ*, yellow), interlayer exciton intensity (*I*_IX_, red), and intensity ratio of interlayer exciton and interlayer trion (*I*_IX_/*I*_IX–_, blue) as the tip‒sample distance changes. When the distance increased to 10 nm, the *Γ* and *I*_X–_ increased, meaning the hot e^‒^ induced *I*_X–_ generation. Reproduced with permission from [140]. Copyright 2023 Springer Nature. **d** Hybridization of dark exciton and defect states. Top: Schematic showing the band structure of WSe_2_ in the K and K′ valleys under different strain levels. The strain for the right panel is 1.2%, and the strain for the left panel is 2.4%. Bottom: Calculated band structures of WSe_2_ under strain of 1.2% and 2.4%. **e** Evolution of PL spectra mapping as a function of strain. When the energy of dark excitons matches either D1 or D2 energy, the oscillator strength shows significant enhancements and the anti-crossing behavior becomes apparent. The strain is about 1.2% and 2.4% for the observation of hybridization with D1 and D2, respectively. Reproduced with permission from [141]. Copyright 2022 Springer Nature. **f** Strain effect on exciton hybridation and gyromagnetic factor. Top: Strain dependence of the energy of coupled direct (*A* exc., red) and indirect exciton (*I* exc., blue) described by the upper (U) and lower (L) branches. Bottom: Measured and calculated *g*-factor as a function of biaxial strain. The yellow circles represent *g*-factors collected in the Bitter magnet. The yellow diamonds represents *g*-factors measured in the superconducting magnet. The solid and dashed lines were obtained under the assumption of *g*_I_ > 0 and *g*_I_ < 0, respectively. *g*_I_ represents the *g*-factor for the indirect exciton. Reproduced with permission from [142]. Copyright 2022 American Physical Society

Recently, numerous studies have investigated excitonic dynamics and exciton-exciton interactions modulated by local strain. An intriguing observation is the exciton-to-trion conversion at the funnelling region in strained TMDC monolayer [22, 23, 60, 139]. Bolotin and co-workers have found that, under high strain condition, the PL spectra collected from the WS_2_ are dominated by the trion emission rather than neutral exciton emission (Fig. 6a, top panel) [22]. In the *n*-doped sample obtained through water and nitrogen desorption, a shoulder peak corresponding to trion emission can be observed even without strain. As the strain is applied, the trion emission becomes the dominant feature due to the reduced Auger recombination and enhanced funnelling efficiency (Fig. 6a, bottom panel). Trion formation arises from the combination of photoexcited excitons with strained-guided electrons, which possesses a conversion efficiency of approximately 100%. The process of exciton funnelling is found to be highly inefficient with less than 4% of photoexcited excitons reaching the center of the funnel, even under the highest achievable strain. The development of tip-enhanced photoluminescence spectroscopic technique has further enabled the visualization of trion formation in 2D TMDC monolayers with nanoscale spatial resolution. For instance, dynamic control of strain with a spatial resolution of 15 nm can be achieved by pressing and releasing a gold AFM tip on a monolayer MoS_2_ (Fig. 6b) [23]. Besides the strain-induced conversion of intralayer excitons, the dynamic control of interconversion between interlayer excitons and interlayer trions can also be realized in heterobilayer structures. By applying the AFM tip on a WSe_2_/Mo_0.5_W_0.5_Se_2_ heterostructure, the interlayer interaction becomes strong due to the reduced layer-to-layer spacing and therefore leads to an increase in interlayer exciton emission intensity. Hot electrons generated from the tip can simultaneously inject into the heterobilayer, giving rise to the formation of interlayer trions (Fig. 6c) [140]. The hot electron injection can be further adjusted to control the interlayer trion conversion rate by precisely regulating the scanning tip distance with a step of ~ 0.2 nm.

Local strain not only changes the electronic band structure but also affects the distribution of energy levels. First-principles calculations reveal that strain induces different energy shifts for different energy bands and their associated valleys [143, 144]. The excitonic species existing in these valleys undergo corresponding energy shifts [145, 146]. The alignment of energy levels between different bands and valleys can be tuned by local strain, resulting in the hybridization of different excitonic states [147–149]. These hybridized exciton states inherit the characteristics of the original states, such as the extended lifetime of dark excitons. They also possess a large oscillator strength, thereby greatly enhancing the light-matter interaction and recombination rate. Recent work has demonstrated the formation of hybridized exciton states in the suspended and deformed WSe_2_ monolayer [141], showcasing enhanced light-matter interaction and strain tunability. The dark excitonic states (X_d_) show a more pronounced response to strain than the local defect states (D1 and D2) (Fig. 6d). When strain is applied to the K and K' band minima, the conduction band strongly shifts downwards while the energy difference between the valence band and defect states remains nearly constant. Consequently, the ${\text{X}}_{\text{d}}^{0}$ state becomes energetically degenerate with the D1 state at ~ 1% strain and with the D2 state at ~ 2.5% strain (Fig. 6d). A remarkable enhancement in dark exciton emission can be detected due to the hybridization between the dark states and the defect-related states (Fig. 6e). Strain enables continuous adjustment of the hybridization between different types of excitonic states and their interaction strength. Furthermore, the coupling of bright exciton and indirect exciton has also been observed with an anticrossing characteristic as the applied strain varied (Fig. 6f, top panel) [142]. Local strain significantly influences the exciton magnetic moment. The gyromagnetic factor (*g*-factor) is observed to decrease under high-strain conditions (Fig. 6f, bottom panel). More effort is still required to elucidate the mechanisms underlying exciton hybridization in strained TMDCs and establish guidelines to manipulate the hybrid excitonic states for novel devices with desired functionalities.

### Phase Transition

**Fig. 7:**
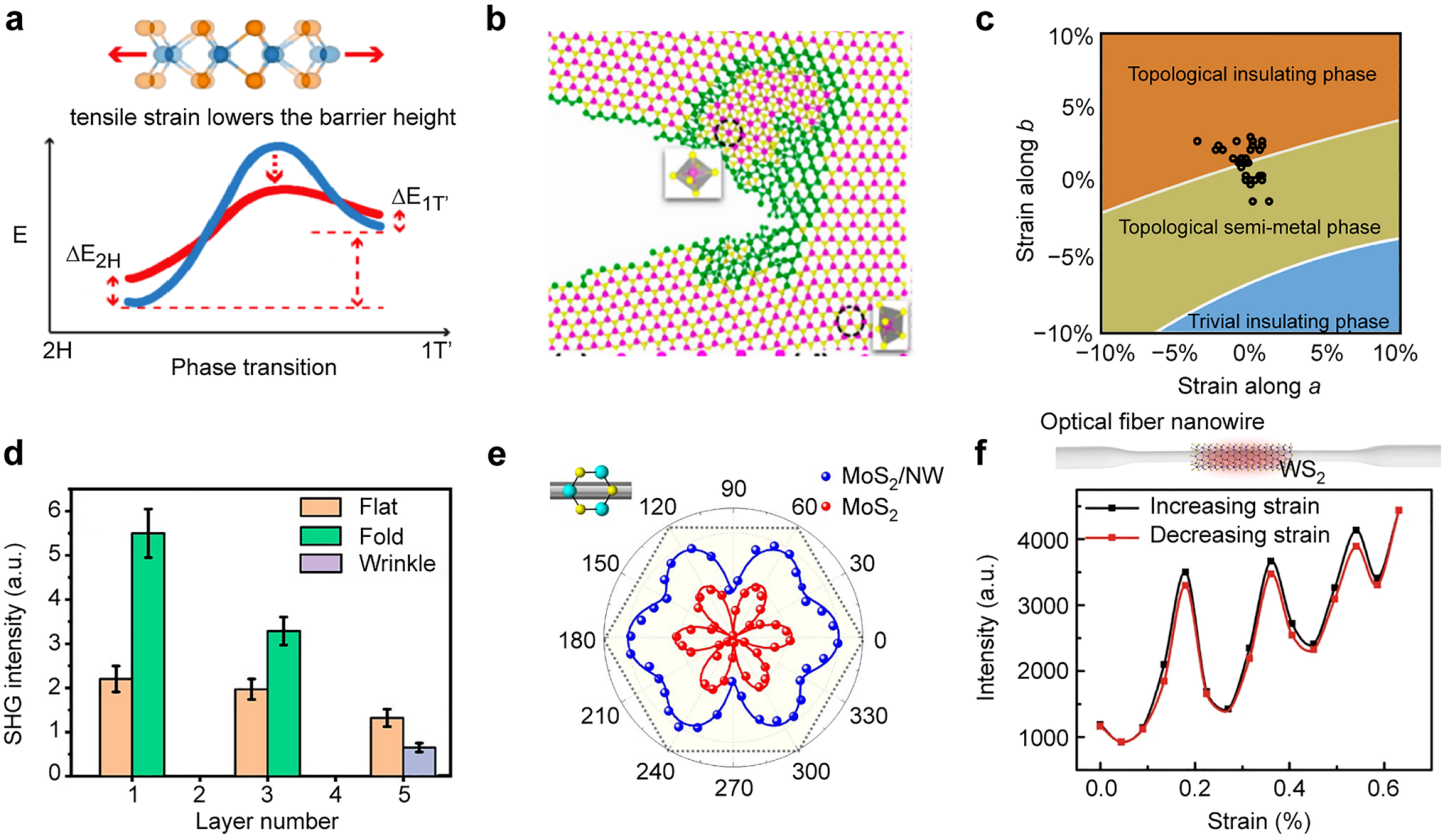
Phase transition and nonlinear optics under local strain effects. **a** Top: Schematic showing the change in atomic structures of MoTe_2_ with tensile strain. Bottom: Barrier energy of the phase transition lowered by tensile strain. Reproduced with permission from [153]. Copyright 2015 American Chemical Society. **b** Schematic illustrating the phase transition from the 2H to 1T′ phase as the crack propagates along the MoSe_2_/WSe_2_ alloy at a strain of 2.15%. Two different crystal structures occur in the displaying region. The green atoms are located at the interface of the 2H and 1T′ phases. Reproduced with permission from [155]. Copyright 2018 American Chemical Society. **c** Phase transition of 1T′ WTe_2_ as a function of strain along *a* and *b* directions. Black circles represent the measured data. Reproduced with permission from [156]. Copyright 2020 American Physical Society. **d** SHG intensity response for the flat, folded (1 layer and 3 layers), and strained wrinkled (5 layers) regions of WS_2_ layers. Reproduced with permission from [157]. Copyright 2020 American Chemical Society. **e** Polar image of the measured SHG intensity from the bare MoS_2_ monolayer (red) and the MoS_2_/TiO_2_ hybrid structure (blue) as a function of the polarization angle of the pump light. The stacking angle between the crystal orientation of MoS_2_ layer and the orientation of TiO_2_ nanowire is 0°. Reproduced with permission from [158]. Copyright 2019 American Chemical Society. **f** Reversible strain manipulation. Top: Schematc of the optical fiber nanowire coupled with a WS_2_ monolayer. Bottom: Measured SHG intensity of WS_2_ monolayer coupled optical fiber nanowires as the function of the strain. Reproduced with permission from [159]. Copyright 2019 Springer Nature

The crystal configurations of TMDCs can be categorized into the 2H phase (trigonal prismatic coordination), 1T phase (octahedral coordination), and 1T′ phase (distorted octahedral coordination) [150]. Among these phases, the 2H phase is thermodynamically the most stable under ambient conditions. The 2H phase typically exhibits semiconducting behavior with bandgap energies ranging from 1 to 2 eV [151], while both the 1T and 1T′ phases tend to exhibit metallic characteristics [152]. The lattice structure and atomic arrangement of TMDCs can also be modified by applying strain, giving rise to the phase transition. A controllable semiconductor–metal transformation has been demonstrated by applying an AFM tip on a MoTe_2_ thin film [153]. This phase transition is driven by two factors: the reduction in the activation energy barrier in the transition state, and the modulation of the cohesive energy of each phase in response to the applied strain (Fig. 7a). Uniaxial strain is effective for inducing phase transitions. Compared to biaxial strain, uniaxial strain can significantly lower the energy barrier for the transition from the 2H phase to the 1T′ phase in MoTe_2_. A uniaxial strain of 6% can cause the phase transition, whereas a biaxial strain of 10% is required [154]. The semimetal-metal transition exhibits complete reversibility under ambient conditions upon the release of strain.

During the CVD fabrication of TMDCs, cracks often occur due to the high stress concentration near precipitate interfaces. Ajayan and co-workers have grown an MoWSe_2_ alloy composed of numerous nanoscopic MoSe_2_ and WSe_2_ regions for investigating the strain effect on mechanical behaviors [155]. In situ Raman measurements and molecular dynamic simulations reveal an irreversible structural transition from the 2H to 1T′ phases around the crack tip. By breaking Mo-Se bonds, the meandering cracks can cross the MoSe_2_/WSe_2_ interface and enter the WSe_2_ patches, resulting in a dramatic change in the tip region. Both the high stress concentration and the biaxial tensile strain can trigger a local structural transformation in irreversible behaviors (Fig. 7b).

In addition to the semimetal-metal phase transition, a semimetal-topological insulator phase transition is theoretically predicted and experimentally confirmed in monolayer 1T′-WTe_2_ [156]. By applying either a compressive strain along the *a*-axis or a tensile strain along the *b*-axis, a phase transition from 1T′-WTe_2_ to a fully gapped insulating phase can be successfully observed (Fig. 7c). With its tunable band structure and stable topology, WTe_2_ serves as an excellent material platform for basic research and topological electronic devices.

### Nonlinear Optics

Nonlinear optical effects manifest when the response of a material to an applied optical field is not linearly proportional to the light intensity. This nonlinear interaction gives rise to diverse optical phenomena, such as sum frequency generation [160–162], second-harmonic generation (SHG) [158], third harmonic generation [162], high-harmonic generation [163], and saturation absorption [164]. The nonlinear response in 2D materials is notably influenced by crystal symmetry, particularly for SHG generation, which is forbidden in centrosymmetric crystals. Owing to the AB stacking order in adjacent layers, 2H TMDCs exhibit opposite crystalline orientations and become noncentrosymmetric (centrosymmetric) in odd (even) numbered layers. As the transition metal atoms are sandwiched between two layers of chalcogen atoms, the breaking of spatial inversion symmetry enables the detection of SHG in TMDCs [165, 166]. Local deformations can effectively tailor the lattice structure by changing the lattice inversion symmetry, and thus modulate the SHG response of 2D TMDCs. About 2–3 time increase in SHG enhancement has been demonstrated in folded WS_2_ region through the mechanical buckling of the flexible substrates (Fig. 7d) [157]. Polarization-dependent SHG measurements enable the estimation of strain amplitude, wrinkle direction, folding angle, and strain vector in wrinkled nanostructures. The integration of MoS_2_ monolayer and TiO_2_ nanowires has been found to enhance the SHG signal due to the broken inversion symmetry by the introduction of TiO_2_ nanowires [158]. Compared with bare MoS_2_ layers, such MoS_2_/TiO_2_ hybrid structures exhibit two orders of magnitude enhancement of SHG signal. Moreover, a significant anisotropy in the SHG signal has also been produced due to the induced directional strain field, which depends on the stacking angle between the nanowire orientation and the crystal orientation of MoS_2_ (Fig. 7e). Similar phenomena have been observed in the integration of monolayer TMDC and an optical fiber nanowire [159, 167–169]. The integration can increase the nonlinear coupling parameter and enhance the power conversion efficiency, resulting in a 20-fold enhancement in SHG intensity [159]. The SHG intensity strongly correlates with the applied strain, showing a periodic characteristic by increasing or decreasing the strain (Fig. 7f). This work presents a promising approach to modulating and regulating SHG by strategically designing and fabricating TMDC-integrated nanostructures.

### Strain Effect on Carrier Mobility and Charge Density Waves

Strain engineering has been established as an effective approach to improve the charge carrier mobility in silicon metal–oxide–semiconductor field-effect transistors in the 1990s. For 2D TMDCs, the strain-induced deformation of the lattice structure can change the location of the conduction band minima or valence maxima in the Brillouin zone [170, 171]. The movement of conduction- or valence-band in the Brillouin zone space can reduce the inter-valley photon scattering [172–174], leading to an enhancement in charge carrier mobility. Owing to the strain-induced band structure modification, the reduced effective carrier mass can also increase the charge carrier mobility [175, 176]. Recent work reported that less than 1% uniaxial tensile strain can double the electron mobility of MoS_2_ [177]. Further simulation and experiment showed that biaxial strain has a stronger effect on the charge mobility of monolayer TMDCs than uniaxial strain [174]. Two-fold higher on-state current and mobility have been achieved in WS_2_ monolayer transistors with 0.3% applied biaxial strain. The electrical characteristics of the fabricated device reverted to their original state following the release of strain. Moreover, local strain in 2D TMDCs can effectively trap and release carriers for the development of memory devices [178].

Charge density waves (CDWs) are a collective electronic phenomenon in condensed matter physics and have been extensively studied in low-dimensional materials. Of particular interest is that CDWs often coexist or compete with superconductivity and magnetism in 2D materials, which gives rise to rich physical properties. [179, 180]. CDWs in 2D materials stem from electron–electron interactions, Fermi surface nesting, and electron–phonon interactions [181]. External stimulus, like temperature [182], electron doping [183], and the local electrical field [184] can influence the phase transition of CDWs. Many studies have shown that strain can also cause changes in the CDW vector and geometry in 2H-NbSe_2_ [185–187]. Two dramatic strain-induced CDW phase transitions have been discovered in 2H-NbSe_2_ due to the sample-substrate mismatch in the thermal expansion coefficient [185]. Two ferromagnetic CDWs can be formed in monolayer VSe_2_ under tensile strain, when accompanied by lattice reconstructions of magnetic V atoms [188]. Unlike the traditional CDWs, these two ferromagnetic CDWs exhibit two distinct half-metallic phases over a large strain range. The strain-modulated CDWs of 2D materials and their underlying physical properties still need to be further explored.

### Theoretical Simulations

Theoretical calculations are important tools for understanding the physiochemical properties of strained 2D TMDCs. As a first-principles quantum mechanical computational method, density functional theory (DFT) can predict the elastic modulus and theoretical strength of diverse 2D TMDCs under applied strain [189, 190]. The results from DFT calculations agree well with the experimental measurements. The electronic band structure, carrier mobility, and surface energy of strained materials can be predicted by DFT calculations [191]. For example, DFT calculations have shown the changes in the band structure of monolayer MoS_2_ under 2% biaxial tensile strain [192]. A 0.46 eV reduction of bandgap and a transition from a direct to an indirect bandgap were predicted. DFT simulations can also visualize strain-induced phase variation of 2D TMDCs [191]. A phase-field model has been established to simulate quite well how phase transition happens and what microstructural features in transformable TMDC monolayer look like [193]. Although DFT methods are generally accurate, they are limited by extremely small simulation cells, which restricts calculations of complex systems with large length scales. More simulation methods are needed to understand the features of strained materials on a large scale.

The molecular dynamics (MD) simulation method is suitable for extending the modelling capabilities to nanometer and micrometer scales. MD simulations can calculate the elastic properties and strength of 2D materials subjecting to different deformations, like wrinkles, uniaxial tension or nanoindentation. As the MD simulation is based on classical mechanics and Newton’s equation of motion, the reliability of the predicted results is highly dependent on the accuracy of the used force fields. Empirical potentials such as Lennard–Jones and Tersoff potential can be used to describe the mechanical properties and deformation behavior of 2D materials [194]. Uniaxial tensile simulations using the Tersoff potential investigate the effects of point defects, grain boundaries, and lattice defects on the elasticity of 2D materials [195, 196]. The Stillinger–Weber potential separately considers bond stretching and angular bending interactions, which is suitable for describing of nonlinear process of strained TMDC system [197]. A disadvantage of this method is that this workflow has limited interatomic potentials available to simulate the mechanics of 2D systems. Only a small number of 2D systems have available interatomic potential. Recently, machine learning offers new opportunities to change the traditional workflow of interatomic potential development by simplifying the process and reducing the reliance on domain expertise [189]. For example, Zhang et al. developed a multi-objective genetic algorithm to parameterize interatomic potentials for large deformation in 2D materials [198].

## Quantum Emitters

Quantum optical technologies have experienced rapid growth, approaching the threshold of a second quantum revolution [199–201]. Quantum emitters, particularly single-photon emitters, play a crucial role as fundamental building blocks for various optical applications, such as quantum communications, quantum computing, and quantum simulation [202, 203]. As mentioned above, local strain exhibits significant promise in manipulating the exciton transport and recombination processes in 2D TMDCs, thereby offering the potential to develop quantum light sources. Here, we review the recent progress in strain-induced quantum emitters with 2D TMDCs.

### Single-Photon Emission in 2D TMDCs

Single-photon emission, distinguishable from classical multiphoton emissuib by its ability to produce temporally and spectrally indistinguishable single photons, has been observed in various TMDCs, initially in monolayers of WSe_2_ [204–207], and later in MoSe_2_ [208, 209], WS_2_ [38, 209, 210], and MoTe_2_ [211, 212]. The single-photon characteristics are assessed by the second-order correlation function at zero time delay *g*^(2)^(0) through Hanbury Brown and Twiss measurement, where the emitted photons are split into two pathways with single-photon detectors to record *g*^(2)^(*τ*) with different time delay *τ*. An ideal single-photon emitter should produce photons one at a time under single pulse excitation, exhibiting photon antibunching, which yields *g*^(2)^(0) = 0. In practical measurements, an emitter is considered a single-photon emitter if *g*^(2)^(0) < 0.5, accounting for the unavoidable occurence of multiphoton emission. Consequently, single-photon purity can be quantified as *P* = 1 ‒ *g*^(2)^(0). To date, the highest single-photon purity measured from TMDCs has reached up to ~ 0.95 and above [26, 213–215].

**Fig. 8:**
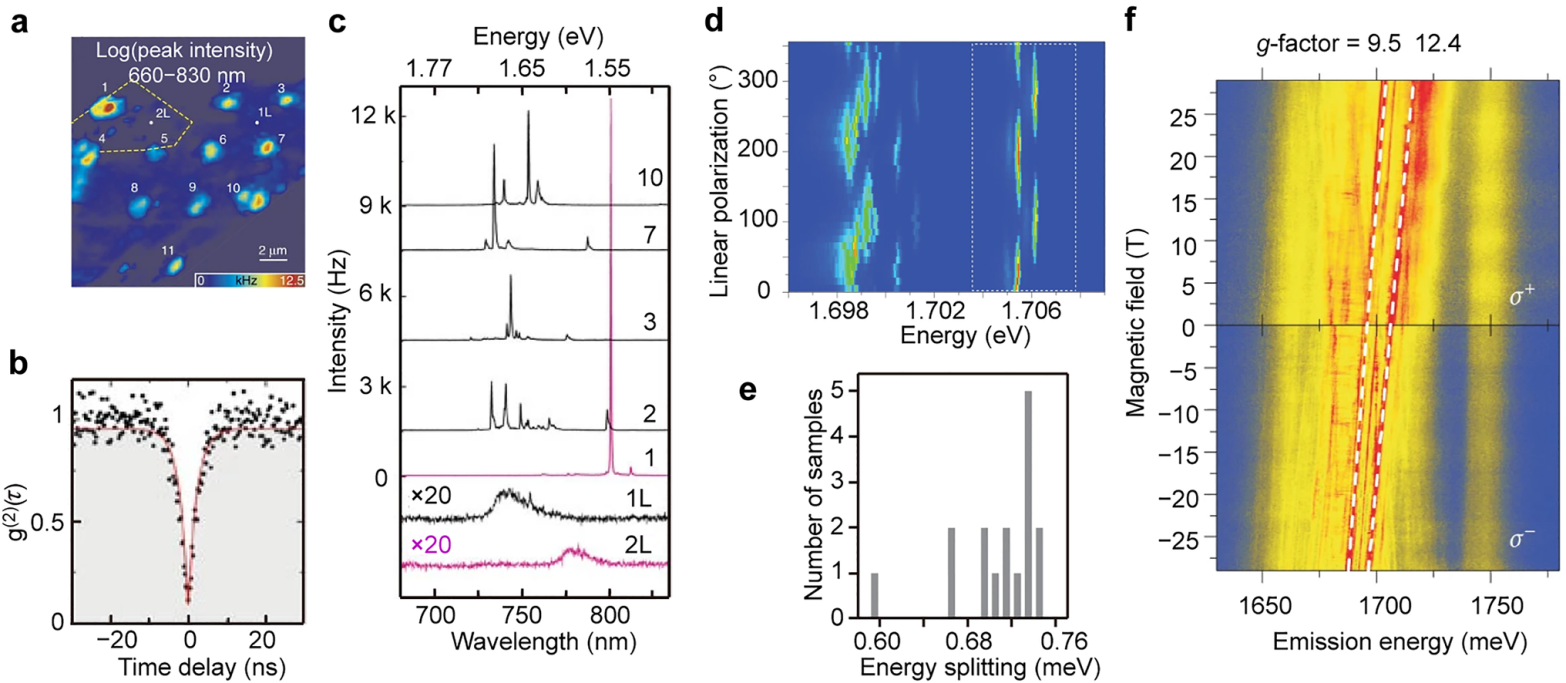
Single-photon emission in 2D TMDCs. **a** Spatially-resolved color-coded PL intensity map in the wavelength range of 660–830 nm. **b** Second-order photon correlation measurement from a representative quantum emitter at a nanopillar location. The red line represents the fitting results with *g*^2^(0) = 0.07 ± 0.04 and *τ* = 2.8 ± 0.2 ns. **c** PL spectra obtained from the indicated nanopillar locations shown in (**a**). The red and black lines with weaker signals correspond to PL spectra collected from unstrained regions of the WSe_2_ monolayer and bilayer, respectively. Reproduced with permission from [26]. Copyright 2017 Springer Nature. **d** PL intensity map of the quantum emitter in a WSe_2_ monolayer as a function of the polarization detection angle and photon energy. Five pairs of cross-linearly polarized spectral doublets are identified. **e** Distribution of the fine structure splitting is measured from 16 individual quantum emitters in the WSe_2_ monolayer. Reproduced with permission from [204]. Copyright 2015 Springer Nature. **f** Magnetic field dependence of PL spectra collected at the edge spot of a WSe_2_ monolayer. The top and bottom panels correspond to the PL spectra resolved in *σ* + and *σ*– circular polarizations, respectively. The extracted *g*-factors of the emission lines from the two quantum emitters are 9.5 and 12.4. Reproduced with permission from [206]. Copyright 2015 Springer Nature

Many TMDCs, particularly in monolayer form, possess a direct bandgap, allowing efficient light emission and absorption. In particular, the presence of K and K' valleys in the Brillouin zone allows for spin-selective transitions [216–218], which is crucial to the generation of circularly polarized single photons for quantum communications and information processing (see more details in Sect. 4.4). Compared to other TMDCs, WSe_2_ exhibits pronounced spin–orbit coupling, which yields the highest exciton binding energy, and it possesses a low-lying long-lifetime dark exciton state that promotes efficient coupling to single-photon emitter states within the bandgap [219, 220]. Therefore, the first a few reports in this field are based on WSe₂ monolayers [204, 206, 207], holding the opinion that these single-photon emission originates from exciton trapped at defects. However, recent works highlight the crucial role of local strain in the formation of single-photon emission in monolayer WSe_2_ [30, 103, 221–223]. For example, 2D WSe_2_ has been integrated with nanopillar-patterned substrates to produce large-scale deterministic local emitters (Fig. 8a) [26]. Second-order correlation measurements conducted at cryogenic temperatures further reveal photon antibunching and the quantum nature of the discrete lines with *g*^(2)^(0) = 0.07 ± 0.04 in monolayer sample (Fig. 8b). The occurrence of single quantum emitters is close to 100% at each nanopillar location. Emissions from these nanopillar locations exhibit inhomogeneous spectral features at energy levels of 10–200 meV below the free neutral excitons, with narrow linewidths reaching as low as 0.1 meV (Fig. 8c). Under resonant laser excitation, the fluorescence linewidth of single quantum emitters can be further narrowed to 0.01 meV [224].

Many of these observed single quantum emitters possess a distinct fine structure characterized by cross-linearly polarized spectral doublets (Fig. 8d) [204]. The exchange splitting between two excitonic eigenmodes is 0.71 ± 0.04 meV (Fig. 8e), resulting from the electron–hole exchange interaction [225]. This exchange splitting is higher and more uniform compared to that of typical III-V quantum dots [226]. Under an external magnetic field, the quantum emission lines exhibit a pronounced Zeeman effect, with *g*-factors ranging from 9 to 12 (Fig. 8f) [206]. This anomalously large Zeeman splitting is likely caused by the inherent strong spin–orbit coupling in WSe_2_, which can be further enhanced by quantum confinement and strain effects [204, 206]. The unique Zeeman splitting characteristics observed in these quantum emission lines offer new possibilities for achieving photon indistinguishability.

Similar strain-activated quantum emitters have been found in other 2D TMDCs. By placing WS_2_ on nanopillars, local strain can be producd, resulting in multiple sub-nanometer spectral lines at each nanopillar location [38]. Moreover, sharp spectral features have been detected at local strain sites in MoSe_2_ [208, 209]. Antibunching measurements further confirm that these sharp emission peaks exhibit single-photon emission characteristics [209]. Quantum emitters in MoSe_2_ are estimated to radiate about 100 times faster compared to those in WSe_2_ and possess a much shorter lifetime of 0.19 ns. Most of the quantum emitters discussed above cover a broad wavelength range from 600–800 nm. The utilization of MoTe_2_ flakes onto nanopillar arrays can extend single-photon emissions to the telecom wavelength band from 1080 to 1550 nm [211]. In MoS_2_, the formation of single-photon emitters is associated with defects introduced by helium ion beam or ultraviolet irradiation, rather than local strain sites [227]. The quantum emitters formed by unpassivated sulfer vacancies possess higher energy levels, ranging from 1.7 to 1.88 eV. These emitters created by irradiation have a lifetime exceeding 1 μs and can be positioned deterministically [228], with the potential for further control by gating [229].

The dangling-bound-free surfaces and weak interlayer van der Waals interaction of 2D TMDCs allows them to stack layer-by-layer, enabling unprecedented properties and applications with the construction of van der Waals heterostructures [230, 231]. In general, when two TMDC monolayers are stacked vertically, the misalignment of rotational angle and lattice constant leads to the generation of the moiré superlattice. For long-range moiré patterns, an array of localized potential wells for excitons can be formed with a series of narrow emission lines. The quantum nature of moiré excitons in heterostructures was first experimentally demonstrated in MoSe_2_/WSe_2_ vertical heterostructures with a rotation angle of 60° [232]. Compared to the single-photon emitters formed in monolayer systems, significantly larger permanent dipoles of interlayer excitons were detected in the vertical direction. These vertical dipoles facilitate the tuning of the quantum emitter energies via the direct current Stark effect [233]. Similar to monolayers, TMDC heterostructures are sensitive to external strain. The interlayer exciton energy can be modulated with the deformation of the 2D layers by using AFM tips [21] or nanopatterned surfaces [234]. The nanoscale deformations caused by the defects can distort the moiré patterns, producing linear polarization and stronger moiré exciton emissions [232, 235].

2D TMDCs exhibit large exciton-binding energies and spin–orbit coupling, leading to the spin-valley degree of freedom [236]. Such strong spin–orbit coupling induces spin-splitting in both conducting and valence bands, giving rise to spin-valley locking. This allows different excitonic valleys to be separately addressed and detected with different optical circular polarizations [237]. One work has demonstrated that spin-valley locking is broken in the single-photon emitters arising from the intervalley defect excitons. Local strain modifies the excitonic energy levels and shifts them into the band gap where they overlap with localized intragap defect states. This overlap facilitates hybridization between the strain-induced local excitons and defect states [148]. These intervalley defect excitonic states exhibit enhanced transition strengths and extended radiative lifetimes. Owing to the spin-valley unlocking, the polarization and energetic position of the single-photon emitters can be significantly changed under applied magnetic and electric fields [148, 204]. Quantum emitters arising from the moiré excitons trapped in a periodic potential landscape inherit the valley-contrasting properties of the monolayer counterpart [28]. For example, moiré-trapped interlayer excitons in TMDC heterobilayers exhibit strong valley polarization with highly uniform *g*-factors, which shows a strong dependence on the layer twist angles [44, 233, 238]. This uniformity of the *g*-factors in the heterobilayers contrasts with that of the excitons bound to defects or strain-induced potentials in the monolayers, where the *g*-factors exhibit a wide distribution across the given sample [204]. Spin-layer locking of interlayer excitons trapped in moiré superlattices has been observed in a heterostructure of bilayer 2H-MoSe_2_ and monolayer WSe_2_ [238]. The spin-locked electron spin and layer pseudospin lead to two quantum-confined interlayer species with distinct spin–layer–valley configurations. Owing to the large permanent dipole moments of interlayer excitons, a tuning range of 40 meV in the single-photon emission energy has been achieved under the applied out-of-plane electric field via the direct current Stark effect [233], which is much larger than that of monolayer WSe_2_ quantum dots [239, 240].

### Integration of Photonic Nanostructures and Quantum Emitters

Photonic nanostructures, especially optical cavities, can concentrate the electromagnetic field into sub-wavelengths to enhance light-matter interactions [241, 242]. Owing to the layered nature and the deterministic embedding, 2D TMDCs hosting the quantum emitters allow for integration with a variety of photonic nanostructures, including dielectric nanoantennas and plasmonic nanostructures. The combination of quantum emitters and nanostructures has the potential to boost their radiative emission rate through the Purcell effect [241, 243]. Such enhancement can increase photon brightness and coherence, improve overall brightness and collection efficiency, and expand the operating temperature range for single-photon emitters [106, 117].

**Fig. 9:**
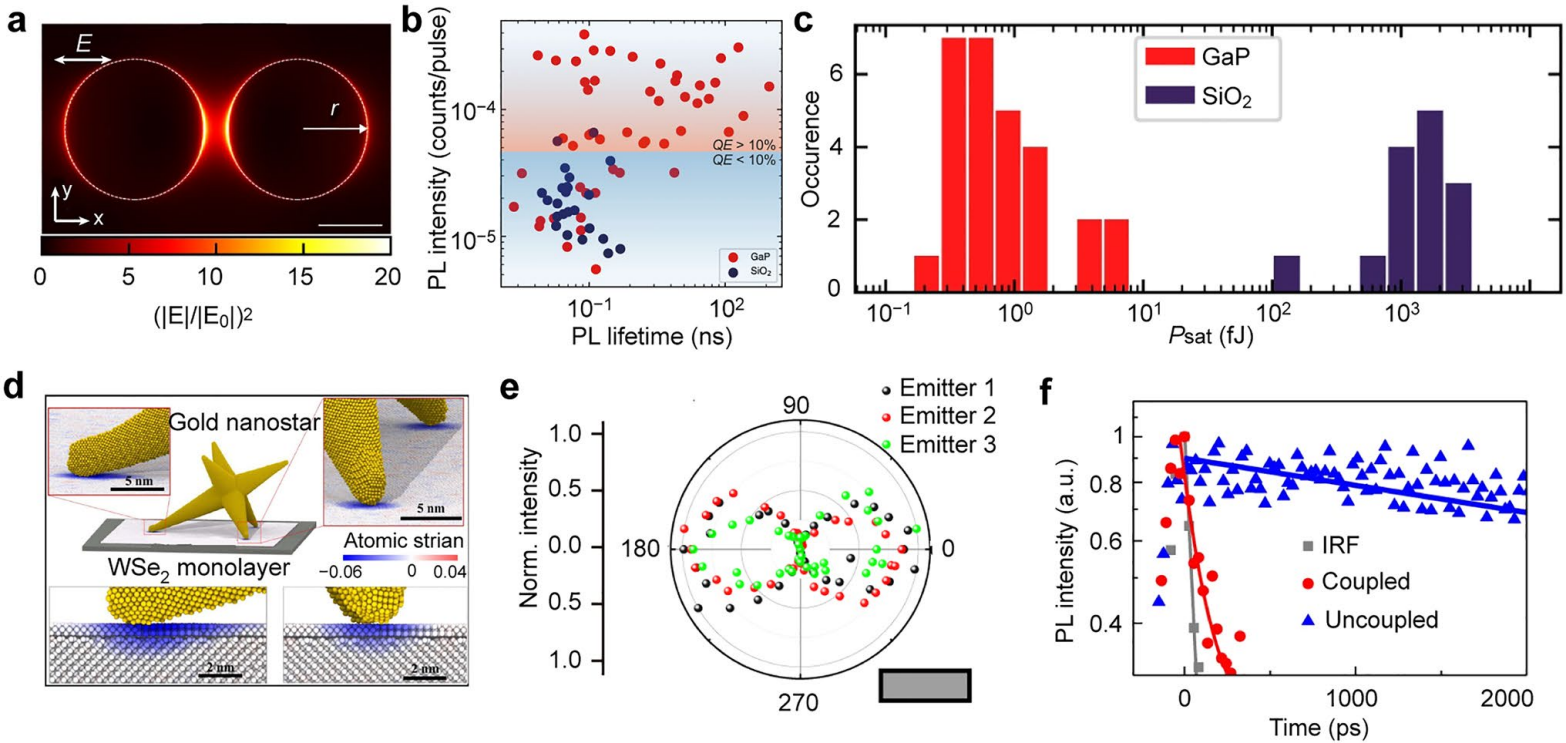
Integration of photonic nanostructures and quantum emitters. **a** Electric field enhancement contours in the *x–y* plane, with the excitation along the *x*-axis. The diameter and the height of each GaP nanopillar are 150 and 200 nm, respectively. The spacing between two nanopillars is 50 nm. Scale bar: 150 nm. **b** Relationship between the decay time and the PL intensity in every pulse. The red and blue spots correspond to the data collected from the single photon emitters formed on GaP dimer nanoantennas and on SiO_2_ nanopillars, respectively. The red and the blue regions represent the formed emitters with quantum efficiency > 10% and < 10%, respectively. **c** Relationship between the occurrence of an emitter and the excitation power density. A pulse energy of 1fJ corresponds to the energy density per pulse of 30 nJ cm^−2^. The single-photon emitters coupled with GaP dimer nanopillars can be observed at low excitation power. Reproduced with permission from [117]. Copyright 2021 Springer Nature. **d** Molecular dynamics simulation of the strain field in a WSe_2_ monolayer induced by a gold nanostar. The top panel is the atomic model. The inset images show the contact region between the WSe_2_ monolayer and the arms of the gold nanostars. The bottom panels are the cross-section views of the inset region from the top panel. Reproduced with permission from [111]. Copyright 2020 American Chemical Society. **e** Polar plot of the polarization features of three individual quantum emitters collected from the rectangular nanopillars. Reproduced with permission from [110]. Copyright 2018 Optica Publishing Group. **f** Measurements of the spontaneous emission lifetime in coupled and uncoupled states. The solid gray line represents a mono-exponential fit with a system response of 65 ps. The grey squares represent the instrument response function for back-reflected laser light. Blue triangles indicate results for the isolated quantum emitter without the cavity. Red circles represent results for the same emitter coupled to the nanocavity. Reproduced with permission from [39]. Copyright 2018 Springer Nature

To improve the quality of the quantum emitters, one effective approach is to combine the 2D TMDCs with high-refractive-index dielectric nanoantennas that possess confined optical modes [33, 102, 117]. These nanoantenna structures can effectively reduce non-radiative losses and significantly increase the radiative rate of the emitting materials [26, 38, 112, 244]. For example, the coupling of a monolayer WSe_2_ with GaP dielectric nanoantennas has been demonstrated to achieve both optical enhancement and strain deformation, enabling the production of single-photon emitters with enhanced quantum efficiency [117]. The PL intensity of the emitter formed on GaP nanoantennas is 10^4^ times brighter compared to the WSe_2_ monolayer placed on low refractive index SiO_2_ pillars. This phenomenon can be attributed to three factors. Firstly, the enhanced adsorption rate enlarges the proportion of excitons participating in emission. Secondly, the Purcell effect accelerates the radiative emission rate, thus enhancing the quantum efficiency. The single-photon emitter can be strategically positioned in regions of significant electromagnetic field enhancement for boosting the radiative rate (Fig. 9a). Lastly, the non-radiative decay can be suppressed in the presence of the crystalline GaP nanoantenna. The resutling single-photon emitters exhibit high quantum efficiency with PL decay time greater than 10 ns (Fig. 9b). The enhanced quantum efficiency allows for operation at low excitation powers (Fig. 9c). In contrast, emitters constructed from WSe_2_/SiO_2_ nanopillars exhibit low quantum efficiency and brightness, necessitating high pumping power for single-photon emissions. However, increased pumping power usually leads to the negative effects, such as Auger recombination, which reduces population inversion. Further improvements in single-photon emitters can be achieved by deterministic defect positioning [30, 228] and advanced growth techniques for high-quality WSe_2_ [39]. Additionally, optimizing the geometric configurations and material compositions of photonic nanostructures holds the potential to improve quantum efficiency.

Plasmonic metal nanostructures with LSPRs provide a powerful means to interact with the light and concentrate it into nanoscale regions. The radiation of single-photon emission can be greatly enhanced by integrating with plasmonic nanostructures due to their substantial field enhancements [28, 32, 110, 110, 237, 245, 246]. Owing to the dimensional merits of plasmonic nanostructures, the strain field can effectively concentrate in nanoscaled regions. By adjusting the geometry and size of plasmonic nanostrutures, both the strain gradients and the plasmon resonance wavelength can be tuned to modulate single-photon emissions. The synergistic effect of strain localization and electromagnetic enhancement helps develop high-performance quantum light sources with plasmon-coupled TMDC nanostructures. For example, gold nanostars deposited on TMDC monolayers can introduce highly local strain fields, achieving a maximum strain amplitude of 6% (Fig. 9d) [111]. The local electromagnetic fields generated in the tip region of these gold nanostars can increase the emission intensities, creating optimal conditions for forming quantum emitters with high spatial resolution. Because of the anisotropic deformation of 2D TMDCs, the local excitons in these structures exhibit polarization that can be controlled by altering the shape and size of the structures [105, 245, 247]. When gold rectanglar nanopillars are covered by a WSe_2_ layer, directional strain is generated and distributed along the horizontal orientation of each nanopillar. Consequently, PL emission is enhanced along the long axis of the gold rectangles (Fig. 9e) [110]. This configuration allows for a remarkable degree of linear polarization through the formation of strongly aligned dipoles with the emitters. Moreover, coupling with plasmonic nanocavities can lead to a significant increase in the radiative decay rate of the quantum emitters [39, 106, 111]. For instance, in a coupled system composed of monolayer WSe_2_ and plasmonic Au nanocube cavity arrays, the emission rate of a representative quantum emitter in the coupled state is 57-fold enhanced compared to the uncoupled state (Fig. 9f) [39]. The quantum yield of the emitters could be improved from 1% to 65% as the flux-grown WSe_2_ combined with the nanocavity. In addition, a few recent works have explored TMDCs coupled to various plasmonic nanostructures, such as plasmonic waveguides [34, 248], single nanowires [40], porous metallic networks [249], enabling the plasmonic single-photon emitters with different configurations.

### Engineering of Quantum Emitters

Owing to low confinement potential and poor quantum yield, quantum emitters with ultra-sharp emission peaks are typically only observable at low temperature below 10 K [32, 111, 205–207, 212, 250]. The integration of quantum emitters with photonic nanostructures effectively enhances the quantum yield, allowing for the observation of single-photon emission at elevated temperature of up to 160 K [30, 106, 214]. However, further advancements are needed to achieve even higher operating temperatures while maintaining stable and efficient single-photon generation, which is advantageous for the development of room-temperature quantum devices [228, 237]. Recent theoretical and experimental studies have highlighted the potential of strain-localized room-temperature emitters as a promising solution to address this challenge [30, 31, 56, 103, 222].

**Fig. 10:**
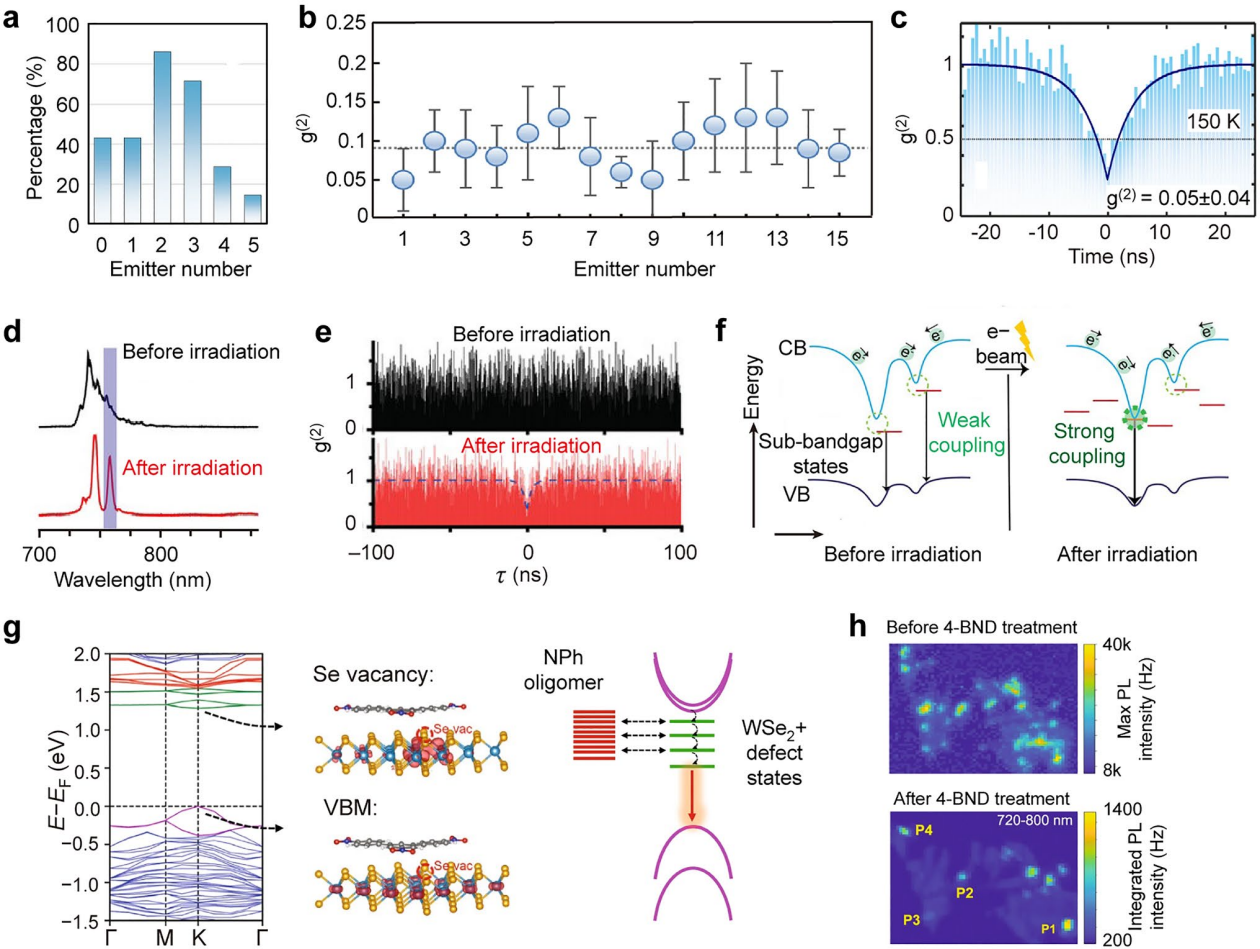
Engineering of quantum emitters. **a** Histogram showing the distribution of emitter numbers measured at high irradiation power of 10^6^ electrons μm^−2^. **b** Distribution of *g*^(2)^(0) for the emitters after irradiation engineering. **c** Second-order correlation function measurement on the emitters at 150 K. Reproduced with permission from [30]. Copyright 2021 Springer Nature. **d** Representative PL spectra for the strained WSe_2_ region before and after e-beam irradiation. **e** Second-order correlation function measurement on the emitters shown in (**d**). **f** Schematic showing the bandgap of the strained WSe_2_ before and after e-beam irradiation. Reproduced with permission from [254]. Copyright 2022 Wiley–VCH GmbH. **g** Band structure calculation for WSe_2_ monolayer with adsorbed molecules. Left: Density functional theory calculation for nitrophenyl (NPh) oligomer physisorbed on monolayer WSe_2_ with a Se vacancy. Right: Schematic illustrating the mechanism for the quenching effect. The black arrows represent the coupling between the NPh orbitals and defect states in WSe_2_. VBM represents the valence band maximum. **h** PL mapping images collected before and after 4-nitrobenzenediazonium (4-BND) treatment. The bottom image involves maximum PL intensity for wavelengths between 720 and 800 nm only. Reproduced with permission from [37]. Copyright 2023 Springer Nature

Surface and interface engineering of 2D TMDC-based quantum emitter has been demonstrated to be an effective strategy for raising the operating temperature of emitters. E-beam and helium ion irradiation has been used to induce structural defects [251–253], mid-gap states [254], and phase transformation [255] in 2D TMDCs. It is important to note that irradiation alone is insufficient for the generation of quantum emitters in WSe_2_. Both strain and defects are found to play a fundamental role in creating single-photon emitters in WSe_2_ [148, 256, 257]. For example, by inducing defects in a WSe_2_ monolayer supported on the nanopillar array, quantum emitters with high yield and purity have been successfully produced [30]. Upon exposure to the higher electron irradiation intensity of 10^6^ electrons μm^−2^ (N_2_), 85% of the sites have exhibited the occurrence of at least one quantum emitter (Fig. 10a). To decouple the strain and defect engineering, e-beam irradiation is first employed to create a wide range of defect energy levels, followed by applying local strain to tune the emission energies of the single-photon emitters. The resulting emitters exhibit an average *g*^(2)^(0) as low as 0.08, representing an average purity of 92% (Fig. 10b) and a linewidth of 75 meV [30]. The temperature dependence of *g*^(2)^(*τ*) measurement indicates that the single-photon nature can preserve the temperature up to 150 K (Fig. 10c). The deep confinement of defects and high activation energy for dissociation contribute to the enhanced performance of WSe_2_ quantum emitters at elevated temperature. These findings provide insights for engineering approaches to achieve room-temperature single-photon emitters, especially for the combination with Purcell-enhanced designs.

Another commonly observed phenomenon in the quantum characteristics of WSe_2_ is the existence of a complex low-temperature spectrum with multiple emission lines [24, 207, 258]. The sub-bandgap photon emission from the defect landscape within the samples contributes to the spectral complexity [258, 259]. Such emissions not only contribute to spectral overlap but also pose challenges in shielding against interference from neighboring emitters and background defects, thereby limiting the single-photon purity. E-beam irradiation on the strained areas has been demonstrated to be an effective way to transform spectrally overlapped sub-bandgap emissions into isolated quantum emission lines [254]. A few distinct peaks can be observed after e-beam irradiation, with the peak number decreased by an average of 44% in the 720–800 nm range (Fig. 10d). The emission from strained areas exhibits anti-bunching behavior (Fig. 10e) and becomes brighter compared to the unstrained regions. E-beam irradiation can induce the additional mid-gap states, thereby increasing the likelihood of single-photon emitters. In highly strained WSe_2_, excitons can funnel into the mid-gap states and strongly couple with the strain-induced minimum conduction states. This mechanism leads to an increase in the number of strong coupled single-photon emitters while reducing the number of weakly coupled emitters (Fig. 10f). In addition to the e-beam irradiation, alternative approaches including plasma treatment [260], helium ion irradiation [253], and site-selective doping [261] have been developed to generate mid-gap states. Moreover, to quench the emission from most strain-induced defect is a common strategy to achieve energetically isolated emitters. Chemical functionalization is a versatile method to tailor the electronic, optical, and excitonic properties of 2D TMDCs [262, 263]. By using molecules to physically adsorb onto WSe_2_, the chemomechanical modification can facilitate energy transfer between molecules and WSe_2_, and therefore quench emissions from the defect states (Fig. 10g) [37]. Despite this, additional defect states with energies lower than the molecular orbitals may persist as the primary source of emission, resulting in the generation of energetically isolated emitters with high purity (Fig. 10h). The integration of molecules and 2D TMDCs in chemomechanical strategy can further enhance the optical properties of the emitters. By selecting molecules with well-aligned energy levels, the selectivity of the emitter wavelength can be refined. Both e-beam irradiation and chemomechanical treatment typically rely on the simplification of defect energy landscape for deterministic emission. However, a significant challenge that remains is exerting more precise control over the defect energy levels to further optimize the properties of these quantum emitters.

### Chirality of Quantum Emitters

Broken inversion symmetry and strong spin–orbit interactions in TMDCs give rise to the formation of spin-valley locked states [264, 265], where excitons in different valleys can be selectively excited due to the optical selection rules [266, 267]. Open questions persist regarding the valley properties inherited by locally trapped excitons and the chirality of quantum emitters under external fields. Despite the progress made in this field, it remains challenging to generate circularly polarized single-photon emitters with high purity and a high degree of polarization.

**Fig. 11:**
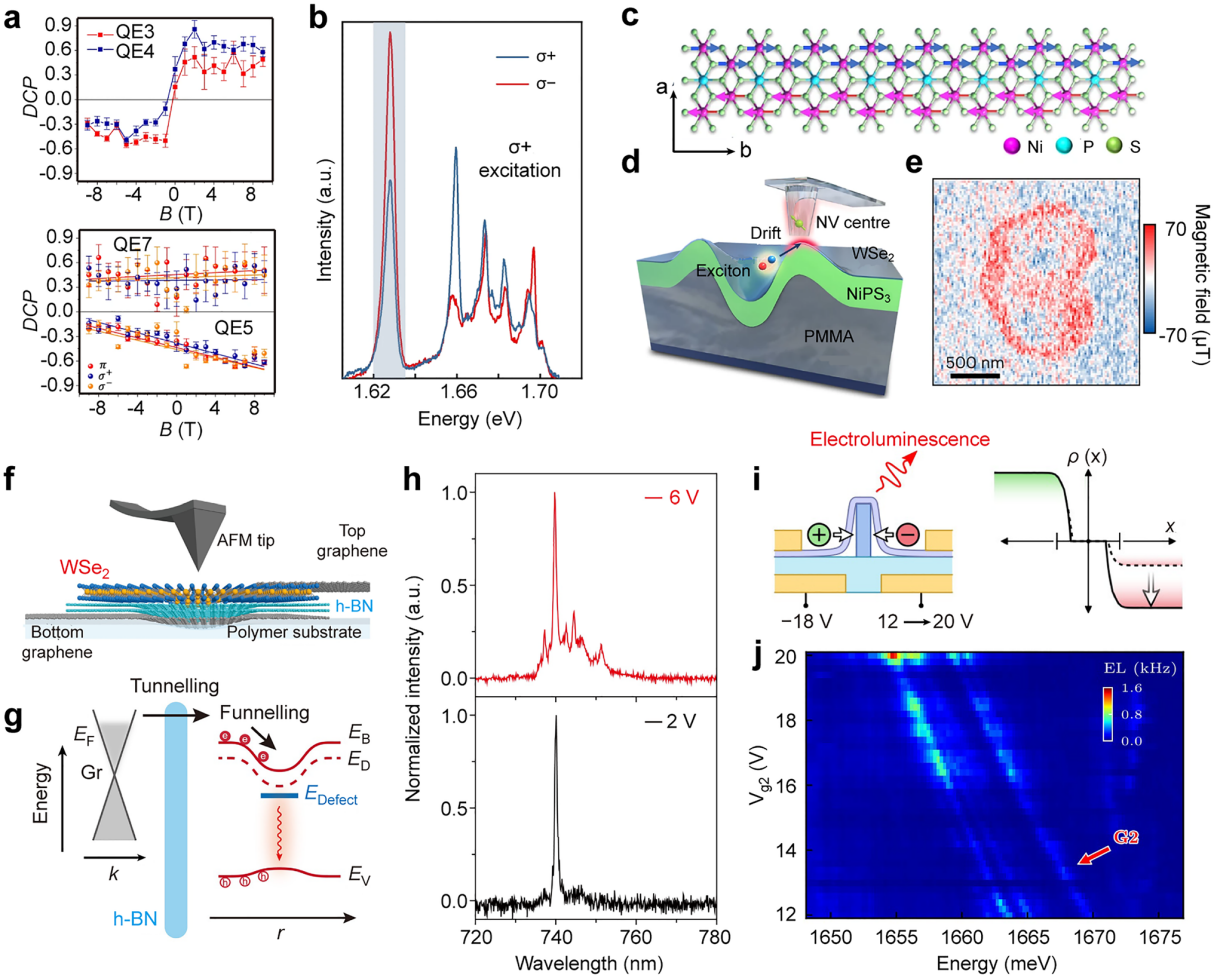
Chirality and electrical excitation of quantum emitters. **a** Degree of circular polarization as a function of magnetic field recorded from different quantum emitters. Reproduced with permission from [269]. Copyright 2023 Springer Nature. **b** Circular polarization-resolved PL spectra collected from an indentation under the excitation of *σ* + polarization. **c** Distribution of antiferromagnetic order in atomic NiPS_3_ structure. **d** Schematic illustrating the cross-section of an indentation region. **e** Magnetic-field image recorded from an indentation. Reproduced with permission from [272]. Copyright 2023 Springer Nature. **f** Schematic diagram of the graphene/WSe_2_/h-BN/graphene vertical heterostructure supported on the polymer substrate. An AFM tip is used to induce indentation in the vertical heterostructure. **g** Band diagram of the deformed heterostructure at an external bias. *E*_F_ represents the Fermi level of the bottom graphene layer. *E*_Defect_ represents the defect band state of WSe_2_. *E*_B_, *E*_D_, and *E*_V_ are the bright excitonic band, dark excitonic band, and valence band state of WSe_2_, respectively. **h** PL spectra collected from the indentation region under the bias voltage of 6.0 V (top) and 2.0 V (bottom). Reproduced with permission from [273]. Copyright 2021 AAAS. **i** Schematic diagram for gate-dependent electroluminescence measurements. The electron density rises with the increase of gate voltage. **j** Electroluminescence mapping collected under the injection current of 5 nA. Reproduced with permission from [274]. Copyright 2022 American Chemical Society

Owing to the asymmetry of the confining potential in TMDCs and circularly polarized exchange interactions in different valleys, a complete circular polarization has been detected in a quantum emitter under a magnetic field perpendicular to the sample plane [24, 205]. Quantum emitters originated from the positive excitons have been demonstrated to possess a valley polarization, which has not been detected in neutral excitons [268]. Recent experimental findings have shown that quantum emitters in WSe_2_ exhibit valley polarization after interacting with a chiral plasmonic nanocavity [269]. An external magnetic field can control the polarization states of the formed emitters (Fig. 11a, QE 3 and 4), which is consistent with the previous studies [205, 270]. However, there are some emitters show almost the same degree of circular polarization with an opposite applied magnetic field (Fig. 11a, QE 5 and 7), propably due to the absence of valley polarization and the coupling with the chiral plasmon field. The local strain-activated quantum emitters exhibit chirality under the external modulation, like combining with antiferromagnetic materials or chiral nanoparticles. Very recently, circularly polarized single-photon emitters have been demonstrated using a strained WSe_2_ monolayer coupled with gold chiral nanoparticles in the absence of an external magnetic field, showing photon antibunching behavior with a *g*^(2)^(0) value of ~ 0.3 [271]. The circular polarization is attributed to the highly localized circumferential current flow on the surface of gold chiral nanoparticles.

Proximity effect, a phenomenon where atomically thin materials acquire properties from adjacent materials through quantum interactions [275–277], has been used to manipulate the quantum emitters in TMDCs [278, 279]. Strain-engineered WSe_2_/NiPS_3_ heterostructures have showcased the capability to modify the chirality of quantum emitters via magnetic proximity interactions [277]. Quantum emissions at indentation sites display a high degree of polarization up to 0.89 even without an externally applied magnetic field (Fig. 11b) [272]. As a kind of antiferromagnetic crystal, the magnetic moments of the Ni atoms in NiPS_3_ are arranged in an antiparallel or parallel manner to the in-plane a-axis, distributing as a zig-zag pattern, which results in a cancellation of the net magnetic moment within the layer while maintaining strong magnetic interactions among the spins (Fig. 11c). Ni atoms at the indentation regions can rotate into the out-of-plane direction due to the deformation of the NiPS_3_ (Fig. 11d). A magnetic signal of 22 ± 9 μT has been measured from the ridge region surrounding the indentation (Fig. 11e). The proximity effect induces the magnetic moments to interact with the local excitons, resulting in the chiral quantum emissions. Similar chiral local excitonic emissions have been detected from other heterostructures like WSe_2_/MnPS_3_ and WSe_2_/FePS_3_ with nanoindentations [272]. Uncovering the physical origin and intrinsic properties of chiral quantum emitters remains a challenge at present. A comprehensive understanding of the underlying physical processes governing the behavior of chiral quantum emitters is crucial for achieving precise manipulation and control of these emitters and their derived devices.

### Electrical Excitation of Quantum Emitters

The ability to electrically pump quantum emitters in quantum nanophotonics is highly desirable, as it enables the scalable solid-state on-chip integration of quantum light sources and the fabrication of compact devices. 2D TMDCs hold advantages as electrically excited quantum light sources as its excitonic behaviors can be readily manipulated by electrical control [24, 229, 280]. Researchers have successfully developed electrically driven quantum emitters with two types of TMDC structures: vertical tunnelling devices and lateral *p-n* junctions [281, 282]. Tunnelling junctions typically consist of graphene as transparent electrodes, hexagonal boron nitride (hBN) as tunnelling barriers, and 2D TMDCs as exciton recombination layers [283, 284]. Although these works prove the feasibility of practical integrated quantum emitter devices, challenges such as indeterminate distributions and inhomogeneous emission energies persist [207, 285]. To create deterministically controlled emission sites, a graphene/hBN/WSe_2_ heterostructure has been integrated with a deformed substrate pattered by an AFM tip (Fig. 11f) [273]. By applying voltage, the Fermi level shifts above the conduction band of the WSe_2_ monolayer, resulting in electron tunnelling through the hBN barrier and enabling narrow electroluminescence emission at the indentation site due to radiative recombination of electron–hole pairs (Fig. 11g). These position-controlled emitters possess the desirable single-photon features with a *g*^(2)^(0) value of 0.3 (Fig. 11h). The properties of local excitons in 2D TMDCs have been proven highly related to the charge density and local field [24, 286]. Gate-defined lateral *p–n* junction devices have been used to manipulate the charge density and field in the junction by modulating the gate voltages while applying a lateral current [274, 287]. By applying positive and negative voltages on the two gates of device, electron- and hole-rich regions can be formed within the WSe_2_ layer, allowing injection of charge carriers into the junction for electroluminescence emission (Fig. 11i) [274]. When the applied voltage reaches to specific value, most of the emission peaks of local excitons are suppressed and a new type of gate-activated emission peaks appears. These gate-activated local exciton peaks become brighter and exhibit a linear redshift of over 10 meV with the increased voltage (Fig. 11j). By adjusting the voltage of the cathode, the energies of gate-activated local exciton states can be modulated, enabling active control of local exciton emission.

## Conclusion and Prospective

In this review, we have provided a overview of the recent advancements in local strain engineering of 2D TMDCs with a focus on creation methods, excitonic properties, and quantum applications. We have examined the various techniques employed to induce local strain in 2D TMDCs, including AFM tips, pre-bended elastomer substrates, bubbles, lattice and substrate mismatch, and patterned templates, each with unique technical aspects, controllability, scalability, and strain carriers. We have outlined the effect of local strain on optical properties and excitonic behaviors in 2D TMDCs, such as exciton funnelling and anti-funnelling, hybridization of diverse excitons, and exciton conversion. We have then highlighted the applications of local strain engineering on 2D TMDCs for quantum technologies, emphasizing their potential in single-photon emitters. Several approaches can be applied to modulate the properties of single-photon emitters, resulting in improvements in brightness, purity, and operating temperature. Furthermore, the chirality of quantum emitters formed in strained TMDCs has been discussed. Finally, we have discussed electrically pumped quantum emitters for TMDC-based devices.

The development of experimental techniques to precisely control the magnitude and distribution of local strain is desired to realize the reconfigurability for further investigations and applications. While a variety of methods have been explored to introduce local strain in 2D TMDCs, the fabrication techniques often rely on manual manipulation of exfoliated TMDC samples [48, 100], inherently limiting sample size and productivity. This manual process can result in uncontrollable and unrepeatable strain distributions, leading to inhomogeneous modulation of material properties. More research efforts have been devoted to efficient exfoliated methods for improving the size and surface cleanliness of the 2D TMDC samples [288, 289]. In theory, Tang et al. presented a data set of the critical interlayer binding force and energy of 230 distinct 2D materials through high-throughput first-principles calculations and revealed an approximate linear relationship between these two parameters [290]. This provides important theoretical support to optimize the exfoliated process for improving preparation efficiency and material quality. The effective strain formed on 2D TMDCs is typically smaller than the expected value [45, 291], which may be caused by the low adhesion strength and the mismatched mechanical properties between the substrate and 2D TMDCs. The ability to produce high-quality strained 2D TMDCs in a scalable manner is crucial for their widespread applications in various technological domains. A position-predictable, repeatable, and periodic method is required for local strain generation in 2D TMDCs. One potential avenue is to take the advantage of CVD techniques to prepare large-area strained 2D film [92, 292]. But the degree of achievable strain is limited and the tunability remains relatively poor [120]. Further exploratory efforts are still required to optimize CVD or alternative techniques to fabricate patterned arrays with controlled local strain, which will unlock the potential of strain engineering in 2D TMDCs and enable their integration into quantum-photonic devices, such as single-photon emitters and active spin qubits.

Further investigation into the origin nature of quantum emitters is essential to gain better control over their properties for further enhancing emission brightness, reducing linewidth, and controlling the energy distribution. Although most works demonstrated that local strain plays a significant role in TMDC-based quantum emitters, the scale of strain sources is much larger than the exciton Bohr radius [293]. The origin of quantum emitters should not be solely viewed from the introduction of local strain, but rather as a result of a combination of local strain and intrinsic crystal defect or crystal disorder induced by the environment [28, 148, 215, 237]. Nonuniform strain alters inherent excitonic energy levels, overlapping these states with defect states. This hybridization improves the emission efficiency, resulting in the enhancement of emitter brightness. Recent work has also demonstrated that the combined effect of defects and local strain can improve the purity of quantum emitters and the temperature of single-photon emissions up to 150 K [30]. More effort is required to combine high-resolution PL measurement techniques with strain distribution measurements to achieve spatial imaging at the atomic scale. The potential correlation of the quantum emission location with strain or defects needs to be further elucidated.

Photon indistinguishability is a critical prerequisite for the successful implementation of various quantum technologies. Even minor differences between individual single photons can disrupt the quantum coherence, rendering associated quantum technologies inoperable. The ideal Hong-Ou-Mandel interference has not been observed in TMDC-based single-photon emitters [28, 236, 294]. Most TMDC-based single-photon sources exhibit significant inhomogeneous broadening with their spectral linewidths exceeding the intrinsic value by orders of magnitude. The undesirable inhomogeneity is attributed to the intrinsic or interface disorder within the TMDC materials, which can give rise to single-photon emission energy fluctuations [259]. These effects are detrimental to the realization of indistinguishable single-photon emitters. One potential strategy is to leverage the Purcell effect to decrease the emission lifetime and thereby narrow the linewidth [295, 296]. Another approach is to mitigate the environmental charge noise from the environment through encapsulation techniques [295]. Unlocking the spin properties and achieving coherent single-spin manipulation in TMDC-based single-photon emitters are critical advancements for quantum information technology. While the spin coherence control in TMDC-based emitters remains unrealized experimentally, the inherent spin-selective optical selection rules present a promising avenue for realizing spin-photon interfaces. The coupling between the electron spin and orbital angular momentum in TMDC materials can affect the spin states, thereby facilitating the realization of coherent spin manipulation. More effort is needed to investigate and understand the spin characteristics of TMDC-based single-photon emitters fully.

Twisted bilayer TMDCs exhibit interlayer excitons, which offer optically addressable spin and valley degrees of freedom with extended lifetimes, enabling potential applications in (pseudo)spin-based computation and fundamental bosonic interaction studies. After successfully constructing quantum emitters trapped in moiré patterns [233], superlattices with relatively long periods provide an intriguing platform for studying interference between different superlattices as well as for creating arrays of quantum emitters. Previous results have demonstrated that different local deformations can contort moiré patterns and introduce new optical selection rules [232]. Moiré superlattices can be visualized with the help of advanced near-field microscope techniques [297]. This allows us to investigate how to vary local deformation with both the selection rule and the transport features. Another area of significant interest is the integration of TMDCs with magnetic materials to construct twisted heterostructures, which provides a viable means to effectively modify valley states of interlayer excitons for practical valleytronic devices. Twisted van der Waals materials offer novel methods for manipulating and controlling quantum materials, creating exciting opportunities for the realization of “quantum matter on demand”. For readers interested in delving deeper into this topic, we recommend referring to another comprehensive review for a detailed exploration of the recent advancements, device architectures, and engineering techniques for tailoring the properties of interlayer excitons [298, 299].

In addition to quantum emitters, 2D TMDCs have also been demonstrated promising potential in photonic devices [300, 301], electronic devices [302], photodetectors [303], energy storage [304], lasers [305], and catalysts [306]. For example, Yang et al. have developed rare-earth doped WS_2_ hybrid structures for an infrared photodetector with high photoresponsivity and detectivity [307]. This hybrid system has shown the potential for advanced photoelectrochemical applications because of the improvement of light absorption and a significant decrease in resistance [306]. One of the primary challenges in utilizing pristine 2D materials for photonic and optoelectronic applications is to improve the exciton dissociation efficiency, which determines photovoltaic conversion efficiency in solar cells and response speed in photodetectors. Local strain engineering presents a promising strategy to modify electronic band structures, thereby influencing exciton binding energy and transport properties. A deep understanding of the exciton radiation behavior and related optical response under applied strain is essential for optimizing devices [308]. Moreover, the deformed 2D TMDCs typically exhibit uneven or irregular morphologies that contribute to a high specific surface area. The combination of high specific surfaces and strain-tuned electronic properties enables local strained 2D materials to be promising candidates for advanced electrocatalytic applications [309].
